# Association of serotonin system-related genes with homicidal behavior and criminal aggression in a prison population of Pakistani Origin

**DOI:** 10.1038/s41598-021-81198-4

**Published:** 2021-01-18

**Authors:** Muhammad Imran Qadeer, Ali Amar, Yung-Yu Huang, Eli Min, Hanga Galfalvy, Shahida Hasnain, J. John Mann

**Affiliations:** 1 Department of Microbiology and Molecular Genetics, University of the Punjab, Khyaban-e-Jamia Punjab, Lahore, 54600 Pakistan; 2 Department of Human Genetics and Molecular Biology, University of Health Sciences, Lahore, Pakistan; 3 Division of Molecular Imaging and Neuropathology, Department of Psychiatry, New York State Psychiatric Institute, Columbia University, New York, NY USA; 4 Mental Health Data Science Division, Department of Psychiatry, Columbia University, New York, NY USA

**Keywords:** Genetics, Molecular biology, Risk factors

## Abstract

The serotonin transporter (*SLC6A4*), 5-HT_2A_ (*HTR2A*) and 5-HT_2B_ (*HTR2B*) recepter genes, express proteins that are important regulators of serotonin reuptake and signaling, and thereby may contribute to the pathogenesis of aggressive criminal behavior. 370 sentenced murderers in Pakistani prisons and 359 men without any history of violence or criminal delinquency were genotyped for six candidate polymorphisms in *SLC6A4*, *HTR2A* and *HTR2B* genes. An association of higher expressing L/L and L_A_/L_A_ variants of the 5-HTTLPR polymorphism was observed with homicidal behavior (bi-allelic: OR = 1.29, *p* = 0.016, tri-allelic: OR = 1.32, *p* = 0.015) and in the murderer group only with response to verbal abuse (OR = 2.11, *p* = 0.015), but not with other measures of self-reported aggression. L/L and L_A_/L_A_ genotypes of the 5-HTTLPR polymorphism were associated with higher aggression scores on STAX1 scale of aggression compared to lower expressing genotypes (S/S, S/L_G_, L_G_/L_G_) in prison inmates. No associations were apparent for other serotonergic gene polymorphisms analyzed. Using the Braineac and GTEx databases, we demonstrated significant eQTL based functional effects for rs25531 in HTTLPR and other serotonergic polymorphisms analyzed in different brain regions and peripheral tissues. In conclusion, these findings implicate SLC6A4* HTTLPR as a major genetic determinant associated with criminal aggression. Future studies are needed to replicate this finding and establish the biologic intermediate phenotypes mediating this relationship.

## Introduction

Aggression and criminal aggressive behavior are a serious concern in most societies worldwide with Pakistan being no exception^1,2^. The extreme forms of aggressive and anti-social behavior include severe violent behavior and homicide. The harm caused by antisocial and criminal behavior includes the victim, their family, society and the perpetrator and their family^3–5^.

The determinants of human aggressive behavior and criminal aggression are complex and include genetic factors and environmental influences (maternal deprivation, violent environment, drug abuse). These effects may be mediated via psychiatric disorders and personality traits. In addition to direct gene or environmental effects, a complex interplay of genes and environment may contribute in the form of Gene × Gene (G × G) and Gene x Environment (G × E) interactions^6–9^. The heritability of antisocial behavioral phenotypes, including criminal and noncriminal aggression, has been estimated to be approximately 50%^10–13^.

Serotonin (5-HT) plays a critical role in the modulation of mood, anxiety, aggression, sleep–wake cycle, motivation, pain perception and neuroendocrine function^14^. Disturbances in the serotonergic system have been implicated in the pathophysiology of several psychiatric disorders including pathological and criminal aggression^15^. Low serotonin turnover rate in brain, as indicated by low 5-hydroxyindoleacetic acid (5-HIAA) concentration in the cerebrospinal fluid, was found in murderers and arsonists and predicts recidivism, and other studies report severity of aggressive behaviors correlate with neuroendocrine evidence of low serotonin function^16^. Moreover, 5-HT_2A_ antagonists may reduce aggression indicating a role for serotonin^17^. Therefore, genes of serotonin system are candidate genetic determinants of antisocial behavior including aggression and criminal aggression^18–20^. Surprisingly few studies have examined the role of serotonergic gene variants in aggression or criminal aggression including homicide^21–23^.

The serotonin transporter gene (*SLC6A4* or *5-HTT*) has several polymorphisms associated with differential expression of serotonin transporter. 5-HTTLPR (serotonin-transporter-linked polymorphic region), was originally reported as a bi-allelic 44 bp insertion/deletion polymorphism in 5′ regulatory region of *SLC6A4*, with a long (L) 5-HTTLPR allele that has a relatively higher gene expression of serotonin transporter than the short (S) allele^24–26^. Additionally, rs25531 in 5-HTTLPR differentiates the L allele into L_A_ and L_G_, where L_A_ mediates a relatively higher transcriptional efficiency of *SLC6A4* as compared with alleles L_G_ and S^27,28^. The 5-HTTLPR polymorphism has been associated with a range of psychopathological conditions including aggression and violent behavior, but with inconsistent results and no clear conclusions as to what allelic combination confers the increased risk^29^.

Serotonin Transporter Intronic VNTR Enhancer (STin2) is a functional 17 bp VNTR polymorphism of human *SLC6A4* gene, and consists of 9, 10 or 12 repeat alleles^30,31^, and the 12 repeat allele has greater gene expression of *SLC6A4*^32^. Similarly, rs1042173 is another functional polymorphism, located in 3′ noncoding region of the *SLC6A4* gene, and may influence the mRNA expression of serotonin transporter^33,34^. Studies of both of these *SLC6A4* polymorphisms report inconsistent results for their association with aggression^35,36^ and different psychiatric disorders^37,38^.

The HTR2A receptor is one of the most abundantly expressed serotonin receptors in the brain^39^, and is located mainly on post-synaptic neurons^40^. The rs6311 single nucleotide polymorphism in the *HTR2A* promoter affects expression^41^. Most studies investigating rs6311 failed to find any significant association with psychiatric traits such as aggression^42^, suicidal behavior^43^ or schizophrenia^44^ although there are exceptions^45^. Similarly, rs17440378 is an intronic SNP in *HTR2B* gene, another important serotonin receptor in the brain, and was recently reported to be a significant genetic determinant of aggression in cannabis-exposed subjects from a Caucasian GWAS study with functional annotations^46^.

Because the most extreme phenotype may be the most informative in terms of genetic associations, the present study investigated the association with homicidal behavior and self-reported aggression of six different polymorphisms in these three serotonergic system-related genes in sentenced prisoners of Pakistani origin.

## Results

The basic characteristics of the study sample set (including demographic details and parameters pertinent to measures of self-reported aggression) are as described previously^47^ and summarized in Supplementary Table S1. Only male prison inmates were included in this study. The mean age of the prison inmates was 36.4 ± 11.8 years, most had a middle class social status (≈72%), a high school education (≈59%) and 56% had been married. The prison inmates and controls were matched demographically [all males and no statistical differences with respect to age (*p* = 0.177), district of origin (*p* = 0.456), socio-economic status (*p* = 0.677) and education (*p* = 0.296).

The primary information (polymorphism, region, type, observed minor allele frequencies, HWE statistics and percentage of samples typed) for serotonergic genes polymorphisms are described in Supplementary Table S2. The allele and genotype frequencies for all studied serotonergic genes polymorphisms did not deviate from Hardy–Weinberg equilibrium in the control group (Supplementary Table S2).

A comparative analysis of allelic and genotypic frequencies in prison inmates and controls for serotonergic gene polymorphisms was done (Table 1). The higher expressing L allele and L_A_/L_A_ genotype of bi-allelic 5-HTTLPR polymorphism and tri-allelic locus of rs25531 in 5-HTTLPR, respectively, were more frequent in prison inmates compared with controls [L *vs.* S: OR = 1.30, *p* = 0.017 and L_A_/L_A_: OR = 1.88, *p* = 0.023 (Table 1)], however, the difference became insignificant after correction for multiple testing. No significant group differences were observed for the allele and genotype distributions of the other serotonergic gene polymorphisms in prison inmates and controls.

**Table 1:** Allele and genotype distribution for 5-HTTLPR, rs25531 in 5-HTTLPR, STin2, 3ʹUTR (*SLC6A4* gene), rs6311 (*HTR2A* gene) and rs17440378 (*HTR2B* gene) polymorphisms and their association with homicide history.

Polymorphisms	Genotype/Allele	Prison inmates, n (%)	Normal controls, n (%)	OR (95% CI)	*p*-value^1^
5-HTTLPR	S/S	111 (30.7%)	128 (36.9%)	Referent	0.046
	S/L	172 (47.5%)	166 (47.8%)	1.19 (0.86–1.66)	
	L/L	79 (21.8%)	53 (15.3%)	1.72 (1.12–2.64)	
	S	394 (54.4%)	422 (60.8%)	Referent	0.017
	L	330 (45.6%)	272 (39.2%)	1.30 (1.05–1.60)	
rs25531 in 5-HTTLPR	S/S	111 (30.7%)	128 (36.9%)	Referent	-
	S/L_G_	38 (10.5%)	41 (11.8%)	1.07 (0.64–1.78)	0.789
	S/L_A_	134 (37.0%)	125 (36.0%)	1.24 (0.87–1.76)	0.238
	L_G_/L_G_	8 (2.2%)	8 (2.3%)	1.15 (0.42–3.17)	0.783
	L_A_/L_G_	27 (7.5%)	18 (5.2%)	1.73 (0.91–3.31)	0.098
	L_A_/L_A_	44 (12.2%)	27 (7.8%)	1.88 (1.01–3.23)	0.023
rs25531 in 5-HTTLPR (genotypes grouped based on relative *SLC6A4* expression)	S/S, S/L_G_, L_G_/L_G_ (Low *SLC6A4* expression)	157 (43.4%)	177 (51.0%)	Referent	–
	L_A_/S, L_A_/L_G_ (Intermediate *SLC6A4* expression)	161 (44.5%)	143 (41.2%)	1.27 (0.93–1.73)	0.133
	L_A_/L_A_ (High *SLC6A4* expression)	44 (12.2%)	27 (4.8%)	1.84 (1.09–3.11)	0.023
*SLC6A4* STin2	12R/12R	164 (45.7%)	173 (48.7%)	Referent	0.540
	12R/10R	156 (43.5%)	151 (42.5%)	1.09 (0.80–1.48)	
	10R/10R	39 (10.9%)	31 (8.7%)	1.33 (0.79–2.23)	
	12R	497 (70.0%)	484 (67.4%)	Referent	0.291
	10R	213 (30.0%)	234 (32.6%)	1.13 (0.90–1.41)	
*SLC6A4* 3ʹ UTR (rs1042173)	G/G	120 (35.1%)	115 (34.2%)	Referent	0.440
	G/T	138 (40.4%)	150 (44.6%)	0.88 (0.62–1.24)	
	T/T	84 (24.6%)	71 (21.1%)	1.13 (0.76–1.70)	
	G	378 (55.2%)	380 (56.5%)	Referent	0.634
	T	306 (44.8%)	292 (43.5%)	0.95 (0.77–1.18)	
*HTR2A* (rs6311)	C/C	123 (33.9%)	122 (34.0%)	Referent	0.530
	C/T	183 (50.4%)	170 (47.4%)	1.07 (0.77–1.48)	
	T/T	57 (15.7%)	67 (18.7%)	0.84 (0.55–1.30)	
	C	414 (57.7%)	429 (59.1%)	Referent	0.581
	T	304 (42.3%)	297 (40.9%)	0.94 (0.76–1.16)	
*HTR2B* (rs17440378)	C/C	257 (71%)	256 (75.5%)	Referent	0.15
	C/T	96 (26.5%)	80 (23.6%)	1.20 (0.85–1.69)	
	T/T	09 (2.5%)	3 (0.9%)	2.99 (0.80–11.17)	
	C	610 (84%)	592 (87%)	Referent	0.12
	T	114 (16%)	86 (13%)	1.29 (0.95–1.74)	

OR, odds ratio; CI, confidence interval; n (%), frequency.

^1^Bonferroni correction for multiple testing was applied (*p*-value threshold 0.0083). Statistically significant *p*-values (< 0.01) and associated OR values are highlighted in bold.

The association between polymorphisms in serotonergic genes and history of homicidal behavior was also tested assuming different genetic models (Table 2). There was an association of the bi-allelic and tri-allelic (rs25531) 5-HTTLPR polymorphisms with homicide (OR = 1.29, *p* = 0.016 and OR = 1.32, *p* = 0.015) in a log-additive model. However, no association was apparent for other polymorphisms of serotonin-related genes and homicidal behavior under all genetic models analyzed. In addition, pair-wise linkage disequilibrium (LD) and haplotype plot structure analysis for serotonergic polymorphisms demonstrated no significant D́ measures between each pair of serotonergic loci analyzed except for bi-allelic and tri-allelic 5-HTTLPR polymorphisms in *SLC6A4* gene (Supplementary Figure S1). The lack of a broader association of serotonergic polymorphisms with criminal aggression was confirmed by a combined genotype analysis considering all the serotonergic polymorphisms analyzed in *SLC6A4, HTR2A* and *HTR2B* genes. However, when haplotype association analysis was performed considering the strong LD between bi-allelic and tri-allelic 5-HTTLPR polymorphisms in *SLC6A4* gene only according to LD block structure, the L-L_A_ haplotype of bi-allelic and tri-allelic 5-HTTLPR polymorphisms was found to be associated with 1.35 fold increased risk of homicidal behavior (OR = 1.35; *p* = 0.011) (Supplementary Table S3).

**Table 2:** Association of the studied polymorphisms in serotonergic system genes with criminal delinquency assuming different genetic models.

Polymorphisms	Model	Genotypes	Prison inmates, n (%)	Normal controls, n (%)	OR (95% CI)	*p*-value^1^
5-HTTLPR	Dominant	S/S	111 (30.7%)	128 (36.9%)	Referent	0.08
		S/L-L/L	251 (69.3%)	219 (63.1%)	1.32 (0.97–1.81)	
	Recessive	S/S–S/L	283 (78.2%)	294 (84.7%)	Referent	0.025
		L/L	79 (21.8%)	53 (15.3%)	1.55 (1.05–2.27)	
	Log-additive	–	–	–	**1.29 (1.05–1.59)**	**0.016**
rs25531 in 5-HTTLPR (genotypes grouped based on relative *SLC6A4* expression)	Dominant	S/S, S/L_G_, L_G_/L_G_ (Low *SLC6A4* expression)	157 (43.4%)	177 (51%)	Referent	0.042
		L_A_/S, L_A_/L_G,_ L_A_/L_A_ (High *SLC6A4* expression)	205 (56.6%)	170 (49%)	1.36 (1.01–1.83)	
	Recessive	S/S, S/L_G_, L_G_/L_G,_ L_A_/S, L_A_/L_G_ (Low *SLC6A4* expression)	318 (87.8%)	320 (92.2%)	Referent	0.051
		L_A_/L_A_ (High *SLC6A4* expression)	44 (12.2%)	27 (7.8%)	1.64 (0.99–2.71)	
	Log-additive	–	–	–	**1.32 (1.05–1.66)**	**0.015**
*SLC6A4* STin2	Dominant	12R/12R	164 (45.7%)	173 (48.7%)	1.00 (Referent)	0.410
		12R/10R-10R/10R	195 (54.3%)	182 (51.3%)	1.13 (0.84–1.52)	
	Recessive	12R/12R-12R/10R	320 (89.1%)	324 (91.3%)	1.00 (Referent)	0.340
		10R/10R	39 (10.9%)	31 (8.7%)	1.27 (0.78–2.09)	
	Log-additive	–	–	–	1.13 (0.90–1.41)	0.290
*SLC6A4* 3ʹ UTR (rs1042173)	Dominant	G/G	120 (35.1%)	115 (34.2%)	1.00 (Referent)	0.810
		G/T-T/T	222 (64.9%)	221 (65.8%)	0.96 (0.70–1.32)	
	Recessive	G/G-G/T	258 (75.4%)	265 (78.9%)	1.00 (Referent)	0.290
		T/T	84 (24.6%)	71 (21.1%)	1.22 (0.85–1.74)	
	Log-additive	–	–	–	1.05 (0.86–1.28)	0.660
*HTR2A* (rs6311)	Dominant	C/C	123 (33.9%)	122 (34%)	1.00 (Referent)	0.980
		C/T-T/T	240 (66.1%)	237 (66%)	1.00 (0.74–1.37)	
	Recessive	C/C–C/T	306 (84.3%)	292 (81.3%)	1.00 (Referent)	0.290
		T/T	57 (15.7%)	67 (18.7%)	0.81 (0.55–1.20)	
	Log-additive	–	–	–	0.94 (0.76–1.16)	0.580
*HTR2B* (rs17440378)	Dominant	C/C	257 (71%)	256 (75.5%)	1.00 (Referent)	0.18
		C/T-T/T	105 (29%)	83 (24.5%)	1.26 (0.90–1.76)	
	Recessive	C/C–C/T	353 (97.5%)	336 (99.1%)	1.00 (Referent)	0.094
		T/T	09 (2.5%)	03 (0.9%)	2.86 (077–10.64)	
	Log-additive	–	–	–	1.30 (0.95–1.76)	0.096

OR, odds ratio; CI, confidence interval; n (%), frequency.

^1^Bonferroni correction for multiple testing was applied (*p*-value threshold 0.0166). Statistically significant *p*-values (< 0.0166) and associated OR values are highlighted in bold.

The serotonergic gene polymorphisms were also analyzed by stratification of the prison inmates’ data into subgroups manifesting self-reported aggression and histories such as childhood history of abuse, history of parental aggression, parental marital problems, any minor psychiatric problems and substance use disorder (Table 3 and Supplementary Tables S4 A–E). The L/L genotype of 5-HTTLPR was more prevalent in prison inmates reporting more susceptibility to provocation by verbal abuse (OR = 2.11, *p* = 0.015) (Table 3). Also, tri-allelic rs25531 in 5-HTTLPR polymorphism was associated with history of any minor psychiatric problems (OR = 0.26, *p* = 0.045) and *HTR2B* rs17440378 polymorphism with parental marital problems (OR = 1.76, *p* = 0.037) (Supplementary Tables S4D and E). However, no associations were found for other serotonergic polymorphisms in the stratified data analyses. The prison inmate group was further analyzed for potential moderation of genetic susceptibility of criminal behavior by environmental risk factors including current age, age at time of committing murder, region, socio-economic, educational and marital status, and ethnic subgroup or caste. However, no effect of these factors was apparent for any of the serotonergic polymorphisms analyzed (Supplementary Tables S5 A–F).

**Table 3:** Association of 5-HTTLPRpolymorphisms with self-reported aggression, emotional reactivity, related psychopathologies and childhood adversities in sentenced murderers.

Response to measure	5-HTTLPR genotypes (recessive model)		OR (95% CI)	*p*-value
	**S/S–S/L, n (%)**	**L/L, n (%)**		
**Self-reported aggression (Lifetime)**				
Yes	180 (65.7%)	59 (76.6%)	1.71 (0.96–3.07)	0.071
No	94 (34.3%)	18 (23.4%)		
**Provoked by verbal abuse**				
Yes	149 (60.6%)	55 (76.4%)	**2.11 (1.16–3.84)**	**0.015**
No	97 (39.4%)	17 (23.6%)		
**Provoked by physical abuse**				
Yes	148 (59.9%)	51 (70.8%)	1.63 (0.92–2.87)	0.094
No	99 (40.1%)	21 (29.2%)		
**Childhood history of abuse**				
Yes	175 (63.4%)	53 (68.8%)	1.28 (0.74–2.19)	0.379
No	101 (36.6%)	24 (31.2%)		
**History of parental aggression towards future aggressor (murderer)**				
Yes	139 (50.4%)	42 (54.5%)	1.18 (0.71–1.96)	0.516
No	137 (49.6%)	35 (45.5%)		
**Parental marital problems (including divorced and separated with step parents)**				
Yes	72 (25.6%)	20 (26.0%)	1.02 (0.57–1.81)	0.950
No	209 (74.4%)	57 (74.0%)		
**Any minor psychiatric problem**				
Yes	13 (5.3%)	02 (2.9%)	0.53 (0.12–2.38)	0.404
No	232 (94.7%)	68 (97.1%)		
**Substance use disorder**				
Yes	55 (20.7%)	13 (20.7%)	0.83 (0.43–1.62)	0.588
No	211 (79.3%)	60 (82.2%)		

OR, odds ratio; CI, confidence interval. Boldface indicates *p* < 0.05 was considered as statistically significant.

State anger score was also determined for prison inmates sentenced for murder (using STAXI subscale, fifteen items) and was correlated with different genotypes of serotonin system genes (Table 4). Prison inmates carrying L and L_A_ alleles of 5-HTTLPR and tri-allelic rs25531 5-HTTLPR polymorphisms, had higher aggression scores compared with inmates with other (S and L_G_) alleles (*p* = 0.0005 for both polymorphisms). However, mean aggression scores did not differ significantly between genotypes for other serotonergic gene polymorphisms.

**Table 4:** Association of *SLC6A4* 5-HTTLPR, STin2, *5-HTT* 3ʹ UTR, rs25331 in 5-HTTLPR and *HTR2A* rs6311 polymorphism with aggression score in sentenced murderers.

Polymorphism	Aggression Score(Mean ± SD)			*p*-value^1,2^
	MM	Mm	mm	
5-HTTLPR	30.68 ± 13.1	39.19 ± 14.8	42.90 ± 14.7	** < 0.0005**
*SLC6A4* STin2	35.34 ± 14.9	38.26 ± 14.7	41.36 ± 15.6	0.071
*SLC6A4* 3ʹ UTR	36.40 ± 15.4	38.30 ± 15.1	37.63 ± 14.3	0.910
*HTR2A* rs6311	35.86 ± 14.4	37.70 ± 15.4	37.63 ± 14.6	0.756
*HTR2B* rs17440378	37.77 ± 15.2	36.63 ± 14.44	36.44 ± 15.82	0.802
	S/S, S/L_G_, L_G_/L_G_ (low *SLC6A4* expression genotypes)	L_A_/S, L_A_/L_G_ (Intermediate *SLC6A4* expression genotypes)	L_A_/L_A_ (High *SLC6A4* expression genotype)	
rs25531 in 5-HTTLPR	30.35 ± 12.6	41.62 ± 14.6	47.02 ± 13.01	** < 0.0005**^**3**^, < **0.0005**^**4**^, 0.052^5^

M, major allele; m, minor allele.

^1^Bonferroni correction for multiple testing was applied (*p*-value threshold 0.0125). Statistically significant *p*-values (< 0.01) are highlighted in bold.

^2^Recessive model for all the polymorphisms (MM and Mm vs. mm).

^3^S/S, S/L_G_, L_G_/L_G_ *vs.* L_A_/S, L_A_/L_G_.

^4^S/S, S/L_G_, L_G_/L_G_ *vs.* L_A_/L_A_.

^5^L_A_/S, L_A_/L_G_ *vs.* L_A_/L_A_.

A gene–gene interaction analysis was also run using the MDR method for the five polymorphisms of three serotonergic system genes analyzed including the tri-allelic rs25531 in 5-HTTLPR, 3′UTR rs1042173 and STin2 polymorphisms from the *SLC6A4* gene, rs6311 and rs17440378 SNPs from the *HTR2A* and *HTR2B* genes, respectively, with murderer/ normal control status as response. Permutation tests failed to validate any significant 2nd, 3rd or 4th order interactions among these five serotonergic system genetic polymorphisms (all *p* > 0.15), suggesting no significant G x G interaction effects for *SLC6A4, HTR2A* and *HTR2B* genes in the present study.

To further investigate the role of studied serotonergic system genetic markers in criminal aggression, we sought to identify any genotype-gene expression based evidence of functional significance of analyzed SNPs by querying the eQTL data available from the Braineac and GTEx databases. We identified significant eQTLs for serotonin system genes analyzed across different brain regions from the Braineac dataset including SLC6A4*rs25531 in hippocampus (*p* = 6.6 × 10^–4^), SLC6A4*rs1042173 in temporal cortex region (*p* = 1.4 × 10^–3^), HTR2A*rs6311 in cerebellar cortex (*p* = 3.7 × 10^–3^), and HTR2B*rs17440378 in substantia nigra region of the normal human brain (*p* = 5.0 × 10^–4^) as presented in Supplementary Figure S2.

Using the GTEx database, we observed that no eQTL data was available for the tri-allelic rs25531 in 5-HTTLPR polymorphism while rs1042173, rs6311 and rs17440378 SNPs were significantly associated with expression of *SLC6A4, HTR2A* and *HTR2B* genes, respectively, in some peripheral human tissues but not in different regions of the brain except for few suggestive associations that included HTR2A*rs6311 in caudate/basal ganglia region (*p* = 0.05) and HTR2B*rs17440378 in cerebellum (*p* = 2.9 × 10^–3^) as well as in spinal cord/cervical c-1 (*p* = 0.03) regions (Supplementary Tables S6 to S8).

## Discussion

In this study, we investigated genetic association of common polymorphic variants in three serotonin system genes (*SLC6A4, HTR2A* and *HTR2B*) with homicidal behavior and self-reported aggression in Pakistani prison inmates sentenced for violent murder(s). We found: (1) A genetic association of the bi-allelic and tri-allelic 5-HTTLPR polymorphisms in the *SLC6A4* gene with homicidal behavior and criminal aggression but not for other polymorphisms of the three serotonin system genes analyzed; (2) Other measures of self-reported aggression and histories (including childhood history of abuse, history of parental aggression, parental marital problems, and substance abuse/dependence) and environmental risk factors (including current age, age at time of committing murder, region, socio-economic, educational and marital status and ethnic subgroup) had no significant role in moderating association of serotonin system polymorphisms and homicidal behavior/criminal aggression in the present sample set (except for described associations); (3) L and L_A_ alleles of the 5-HTTLPR polymorphism had a dose dependent relationship with greater aggression score in sentenced murderers; (4) no significant G × G interaction effects for *SLC6A4, HTR2A* and *HTR2B* genes in the MDR analysis following the hypothesis that these genes may have a synergistic effect in the development of homicidal behavior/criminal aggression since it is a complex trait, and (5) tri-allelic 5-HTTLPR and other serotonergic polymorphisms as functional eQTLs influencing gene expression in different regions of normal human brain and peripheral tissues as determined by the Braineac and GTEx databases.

We also genotyped our samples for the newer tri-allelic variant of 5-HTTLPR polymorphism where L allele is further divided into L_A_ (higher transcriptional efficiency of serotonin transporter gene) and L_G_ (lower transcriptional efficiency of serotonin transporter gene comparable to the S allele) variants based on rs25531 SNP within 5-HTTLPR polymorphism. This tri-allelic variant of 5-HTTLPR polymorphism is a better indicator of expression differences due to the 5-HTTLPR polymorphism and thereby superior study power^48^. Therefore, the main hypothesis was based on tri-allelic HTLPR variant in the present study and the statistical analysis reported above for bi-allelic HTLPR polymorphism was included only for comparison with other reports as most previous studies have only analyzed bi-allelic version of 5-HTTLPR polymorphism.

Efforts were made to analyze those polymorphisms of serotonin system genes that were reported to have functional relevance to serotonin homeostasis. A critical review of the influence of 5-HTTLPR genotypes on *SLC6A4* promoter efficiency and expression^49^, reported that apart from a couple of studies^50,51^, most studies find that the longer repeat (L and L_A_) variants functionally mediate higher basal and induced transcriptional activity of *SLC6A4* promoter resulting in greater gene expression, higher levels of serotonin transporter and an increased serotonin reuptake activity as compared to L_G_ or S variants^24–28,52–61^. In further support of these findings, less in vivo serotonin transporter mRNA levels are associated with S allele of 5-HTTLPR polymorphism^62–64^. STin2 VNTR polymorphism mediates transcriptional regulation of *SLC6A4* gene expression as shown in transgenic mice and in vitro functional data from embryonic stem cells^32,65^, where the 12 repeat allele (12R) displayed higher enhancer-like activity and greater expression of serotonin transporter mRNA as compared to 10 repeat allele (10R)^54,66^. However, data regarding the functional relevance of *SLC6A4* 3′UTR (rs1042173) variant^34,67^ and *HTR2A* rs6311 polymorphism^68,69^ are somewhat conflicting.

With the availability of modern genetic variant-gene expression databases such as the Brainneac and GTEx, it is possible to evaluate the link between genetic polymorphisms and altered gene expression which may help in more precise understating of connection between GWAS/CGAS reported genetic polymorphisms and molecular pathogenesis of different diseases. Therefore, as an alternative validation strategy, we performed a follow-up functional annotation analysis of different serotonergic system polymorphisms analyzed. We identified a significant eQTL pair for SLC6A4*rs25531 in hippocampus region of normal human brain providing further evidence of its functionality and candidacy as potential genetic determinant of pathological aggression.

We also observed that rs1042173, rs6311 and rs17440378 SNPs were also significant eQTLs of *SLC6A4*, *HTR2A* and *HTR2B* genes, respectively, modulating their expression in some human brain and peripheral tissues, although no association with criminal aggression was apparent for these polymorphisms in our study. It is important to note here that differences in methylation and transcriptome regulation patterns in different brain regions may result in distinct gene expression profiles^70^. Moreover, previously it has been reported that risk effects of *HTR2B* rs17440378 polymorphism on aggressive behavior appear to be cannabis use dependent rather than driven by aggression alone or aggression under the influence of other drugs^46^, which may explain no association observed with criminal aggression in the present study using violent murders sample set and a similar explanation may be extendable to other serotonergic polymorphisms analyzed for which no genetic association was observed in the present study. Overall, a functional regulatory role for rs25531 in *SLC6A4* gene is suggested that may influence the gene expression and risk of criminal behavior. These data also stress the need of direct genetic variant mediated functional studies in target tissues to confirm any potential effects of serotonergic system polymorphisms on relevant protein function and/or regulation.

Some previous studies have examined serotonergic polymorphisms in prison inmates, however, studies examining extreme violence phenotype such as sentenced murderers are scarce especially from Indo-Pak subcontinent. Previously, Cherepkova and colleagues reported significant over-presentation of 5-HTTLPR and STin2 risk alleles and haplotype in convicted subjects and MMA fighters (including those convicted of grave crimes or murder) as compared to control group^71^. In agreement with our results, the L allele was more frequent in aggressive adolescent prisoners of Korean origin as compared to normal non impulsive population controls in an earlier study^72^. L/L genotype of 5-HTTLPR was found to be associated with aggressive and violent behavior^73^, borderline personality disorders^74^ and suicidal behavior^75^. Low heart rate and 5-HTTLPR L/L genotype were associated with higher arrest rates for violence in an incarcerated sample of American men^76^. Other psychopathologies in a violent population, such as depression, were also reported to be associated with both the L allele and L/L genotype of 5-HTTLPR polymorphism in Russian criminal offenders^21^.

Some studies report different results such as an association of the lower expressing serotonin transporter S allele or S/S genotype of 5-HTTLPR polymorphism with extreme criminal behavior in Chinese male prisoners^77^ and violent behavior in adult German criminal offenders with history of childhood ADHD^78^. Other studies also reported association of S allele or S/S genotype with antisocial alcoholism^79^, concussion history and personality traits in rugby players^80^, suicidal ideation in acute coronary syndrome patients^81^, higher impulsivity, hostility and neuroticism in anxiety phenotype^82^, hyperactivity-impulsivity in children moderated by peer problems earlier in childhood^83^ and increased exposure to life stressors moderated by ADHD symptoms early in life^84^. Some meta-analyses also describe association of low expression allele/genotype of 5-HTTLPR polymorphism with anti-social behavior^85^, violent suicide attempts^86^ and bipolar disorder^87^.

Like current study, fewer studies analyzed the tri-allelic variant of rs25531 in5-HTTLPR polymorphism and differentiated between L_A_ and L_G_ alleles while determining association with anti-social behaviors and aggression. The S and L_G_ (low *SLC6A4* expression) variants of tri-allelic rs25531 in5-HTTLPR polymorphism were found associated with higher scores on aggression, and total behavior problems in Mongolian children^88^ and higher neuroticism in healthy Chinese men^89^.A few studies also reported no association between 5-HTTLPR polymorphism and anti-social phenotypes including antisocial alcoholic behavior^90^ and aggressive traits^91^.

The lack of agreement regarding the association of 5-HTTLPR polymorphism with aggressive behavior may be explained by several reasons, including but not limited to: small sample sizes; differences in measures or types of homicidal/criminal behavior and aggression (for example reactive aggression *vs*. pro-active or pre-meditated aggression); differences in genetic background or in allele/genotype frequencies based on ethnic origin; and differences in study design and robustness of data analysis (for example demonstrated conformance to HWE). Also, analysis of limited number of polymorphisms in serotonergic genes has its own limitations as expression of a particular gene can be modulated by numerous such genetic variants which may or may not exhibit linkage disequilibrium. Moreover, which allele of the 5-HTTLPR or tri-allelic rs25531 in 5-HTTLPR polymorphism confers the risk of homicidal behavior and aggression is not clear^29^. Two hypotheses have been proposed in this regard; the L and L_A_ variants of 5-HTTLPR polymorphism (mediating a higher functional expression of serotonin transporter gene) result in greater serotonin reuptake and reduced availability of synaptic serotonin, which may increase susceptibility to proactive aggression and anti-social phenotypes^29,85,92^. In contrast, S and L_G_ alleles of 5-HTTLPR polymorphism (that mediate relatively lower transcriptional efficiency of serotonin transporter gene), due to lower serotonin uptake and higher synaptic serotonin, may result in heightened psychotic or paranoid features and thereby a predisposition towards paranoid reactive aggression phenotypes^29,92^.

Genome Wide Association Studies (GWASs) of aggression and criminal aggression are few. Despite some interesting leads highlighting novel loci and pathways in nervous, neuroendocrine and immune systems, GWASs of aggression and related phenotypes (antisocial behavior, anger, conduct problems and callous, uncaring and unemotional aggressive traits) failed to replicate any findings^93–95^. A GWAS study of violent prisoners in Finland (n = 5,983), the most relevant phenotype to the current study, found suggestive association with a polymorphism (rs11649622) in the *CDH13* gene that codes for a neural adhesion protein^96^. One of the largest aggression GWAS studies (n = 18,988) investigating aggressive behavior in children (the EAGLE consortium) reported significant association with the rs11126630 polymorphism located near the excitatory synapse development gene *LRRTM4*, and using a subsequent “classical candidate gene association analysis” also with *AVPR1A* (arginine-vasopressin receptor 1A) gene^97^. Other GWASs report associations with: (a) *HTR2B* serotonin receptor in cannabis-related aggression^98^; (b) 13 different SNPs associated with violence in schizophrenia^99^; and (c) dopaminergic, glutamatergic and neuroendocrine polygenic risk score (PRS) associated with callous-unemotional traits^100^. We also analyzed the GWAS reported *HTR2B* rs17440378 SNP in our homicidal prison inmate sample set but failed to detect any significant association with criminal aggression. Finally, a meta-analysis of GWAS data from five different population studies did not find any significant markers for anti-social behavior^101^. As in many other conditions, GWASs of aggression and criminal behavior largely failed to detect associations for candidate genes, including serotonin neurotransmission, that are mostly being investigated in candidate gene association studies^94^. Perhaps with increasing sample sizes and more homogenous and in-depth definition of the aggression and criminal aggression, we may find such associations, like other complex traits for which GWAS has been a successful approach^95^.

Study limitations include small sample size due the rare population being studied because we sought a group with an extremely severe phenotype, limited time allowed for interview to obtain all clinical phenotypic data, and analysis of a limited number of serotonergic polymorphisms in a limited number of genes instead of conducting an unbiased GWAS study. Another limitation of our study is the lack of any direct functional assessment of the effects of analyzed serotonin system polymorphisms on relevant protein function, including regulation. Inclusion of information about epigenetic marks and expression profile would have enriched the data set. Therefore, replication studies are needed in larger well characterized samples using a GWAS approach, genome-wide DNA methylation and RNAseq data on the transcriptome combined with additional data on developmental history that would permit estimation of G × G and G × E interactions in relation to extreme aggressive behavior and related endophenotypes.

In conclusion, the L and L_A_ variants of 5-HTTLPR VNTR polymorphism with demonstrated eQTL based functional significance are associated with homicidal behavior and self-reported aggression in this rare sample set of Pakistani prison inmates sentenced for murder(s). Analysis of genetic risk factors in extreme criminal violence may increase understanding of the neurobiological basis of less severe aggression. Further studies should also determine whether these findings extend to less severe aggression and related anti-social endophenotypes.

## Subjects and methods

### Ethical approvals, consent and study subjects

The details of consent, subject recruitment and study sample set have been described previously^47^. Briefly, the present study was conducted after obtaining permission and approval from higher police authorities and Institutional Ethics Committee at University of the Punjab, Lahore and all experiments were performed in accordance with latest version of Declaration of Helsinki guidelines. All the study subjects gave written informed consent after the nature of the research procedures had been fully explained to them.

This study comprised 729 subjects, including 370 men in three major district prisons of Punjab, Pakistan that were sentenced for first-degree murder(s), and 359 age and ethnicity matched control men randomly selected from the general population and found to be without any personal or family history of criminal aggressive behavior. Only those prison inmates who had been sentenced for first-degree homicide(s) by a court of law and/or self-confession of the said crime with alleged/convicted/condemned status, were enrolled in the study. A random sampling procedure was ensured by randomly selecting murderers from different barracks within a prison and recruiting murderers from three different major district prisons (and their sub-jails) in Punjab province of Pakistan. It should be noted that prison inmates, as well as normal controls, who had a DSM-IV diagnosis or family history of any major psychiatric disorder, were excluded from the study. Prison inmates were interviewed to record self-reported aggression and histories based on State Trait Anger Expression Inventory (STAXI), with a cut-off value of > 20 as high aggression score, followed by collection of blood samples for genetic analysis.

### Genotyping of SLC6A4, HTR2A and HTR2B polymorphisms

A modification of standard phenol–chloroform extraction method was used for the isolation of genomic DNA from blood samples^102^. All the subjects were genotyped for four*SLC6A4* and one *HTR2A* polymorphisms as described in Supplementary Table S9. Briefly, specific primers were used to amplify the target region containing the polymorphism of interest in a standard PCR reaction. Amplified DNA fragments were resolved on 2% agarose gels and genotypes were directly scored for polymorphisms with PCR based genotyping approach (5-HTTLPR and STin2 polymorphisms). The *HTR2B* rs17440378 SNP was genotyped using a custom iPLEX SNP genotyping assay and the MassARRAY system according to the standard protocol^103^. The remaining polymorphisms (tri-allelic rs25531 in 5-HTTLPR and, 3′UTR rs1042173 in *SLC6A4* and rs6311 in*HTR2A*) were genotyped using a PCR–RFLP based method where PCR amplification products were subjected to restriction digestion by relevant restriction enzyme and restriction digestion products were run on 3% agarose gels to determine respective genotypes in each sample. The representative gel electropherograms for each of PCR or PCR–RFLP based genotyping assays have been provided in the Supplementary Figures S3 to S6. The genotypes of all polymorphisms analyzed were scored by two independent researchers to minimize bias.

### Statistical analysis

The study data were coded and analyzed using the Statistical Package for Social Sciences (SPSS) version 20 for Windows and the SNPstats program^104^. Hardy–Weinberg equilibrium (HWE) analysis was performed in the control group by means of a chi-square test using the SNPstats program. Minor allele frequencies in the present sample for polymorphisms analyzed were all 5% or above. Allele and genotype frequencies were compared between prison inmates and controls using chi-square tests. Odds ratios (ORs) with 95% confidence intervals (CIs) and associated *p*-value were calculated as a measure of association. SNPstats was also used to determine genetic associations assuming the dominant, recessive and log-additive models by unconditional logistic regression analysis. Combined genotype and haplotype frequencies for *SLC6A4*, *HTR2A* and *HTR2B* polymorphisms were also analyzed and compared. The pairwise linkage disequilibrium analysis for serotonergic gene polymorphisms was conducted using the Haploview program^105^. Bonferroni correction for multiple testing was performed when determining genotype, combined genotype and haplotype associations with murderer phenotype and aggression scores. A *p*-value (two tailed) of less than 0.05 was considered significant unless otherwise stated.

Gene–gene interactions were also tested using the Multifactor Dimensionality Reduction (MDR) method^106^. Briefly, this method aims to reduce the number of cells in 2nd and higher order interactions by grouping them based on their level of risk. In case such as this study where there is a binary outcome, multi-loci genotypes are grouped into low and high risk genotypes, then each is separately tested against other genotype combinations using a Wald test. Significant Wald statistics are then validated using permutation tests. We used the “mbmdr” library in R^107^, with a liberal *p* = 0.10 cutoff for the risk classification selecting the low and high risk genotype combinations, and *p* = 0.05 for the permutation test. Five polymorphisms were entered into the analysis: the tri-allelic rs25531 in 5-HTTLPR, 3′UTR rs1042173 and STin2 polymorphisms from the *SLC6A4* gene, rs6311 SNP from the *HTR2A* gene, and rs17440378 polymorphism from the *HTR2B* gene, and 2nd, 3rd and 4th order interactions were tested. Only subjects with data for all five of the above polymorphisms were included in the analysis (370 sentenced murderers and 359 normal controls).

### Expression quantitative trait loci (e-QTL) analysis

To evaluate the association between SNP based genetic markers of serotonin system genes and their relative gene expression, cis-eQTL data were extracted using two modern databases including the Braineac database from the UK Brain Expression Consortium (UKBEC) available at www.braineac.org, and Genotype-Tissue Expression (GTEx) project dataset version 8 (https://www.gtexportal.org/). The Braineac dataset harbors eQTL data of 10 brain regions from 134 postmortem neuropathologically normal individuals of European origin. While the GTEx database is a comprehensive public resource that contains genotypic and tissue specific gene expression data of samples collected from 54 non-diseased tissue types (including brain tissue) across almost 1000 individuals of primarily Caucasian ancestry.

## Supplementary Information

Supplementary Information.

## Data Availability

The datasets generated during and/or analyzed during the current study are available from the corresponding author on reasonable request.
