# Author Correction: Association of serotonin system-related genes with homicidal behavior and criminal aggression in a prison population of Pakistani Origin

**DOI:** 10.1038/s41598-021-91477-9

**Published:** 2021-06-03

**Authors:** Muhammad Imran Qadeer, Ali Amar, Yung‑Yu Huang, Eli Min, Hanga Galfalvy, Shahida Hasnain, J. John Mann

**Affiliations:** 1 Department of Microbiology and Molecular Genetics, University of the Punjab, Khyaban-e-Jamia Punjab, Lahore, 54600 Pakistan; 2 Department of Human Genetics and Molecular Biology, University of Health Sciences, Lahore, Pakistan; 3 Division of Molecular Imaging and Neuropathology, Department of Psychiatry, New York State Psychiatric Institute, Columbia University, New York, NY USA; 4 Mental Health Data Science Division, Department of Psychiatry, Columbia University, New York, NY USA

Correction To: *Scientific Reports* 10.1038/s41598-021-81198-4, published online 18 January 2021

This original version of this Article contained an error, as the paper did not discuss the related work of Cherepkova et al. (2018). As a result, Reference 71 was omitted and is listed below,

Cherepkova, E.V., Maksimov, V.V. & Aftanas, L.I. Polymorphism of serotonin transporter gene in male subjects with antisocial behavior and MMA fighters. *Transl Psychiatry* **8**, 248 (2018). 10.1038/s41398-018-0298-0

In addition, the text in the Discussion,

“Some previous studies have examined serotonergic polymorphisms in prison inmates, and no study looked at extreme violence such as sentenced murderers.”

Now reads,

"Some previous studies have examined serotonergic polymorphisms in prison inmates, however, studies examining extreme violence phenotype such as sentenced murderers are scarce especially from Indo-Pak subcontinent. Previously, Cherepkova and colleagues reported significant over-presentation of 5-HTTLPR and STin2 risk alleles and haplotype in convicted subjects and MMA fighters (including those convicted of grave crimes or murder) as compared to control group^71^"

The original Article has been corrected.

