# Supplementary material for: Association of serotonin system-related genes with homicidal behavior and criminal aggression in a prison population of Pakistani Origin
Source: Sci Rep. 2021 Jan 18;11:1670. doi: 10.1038/s41598-021-81198-4 (PMC7813852; doi:10.1038/s41598-021-81198-4)
Supplement: Supplementary file 1 — Supplementary Information. [file 41598_2021_81198_MOESM1_ESM.docx]

**Manuscript title**

Association of Serotonin System-Related Genes with Homicidal Behavior and Criminal Aggression in a Prison Population of Pakistani Origin

**Authors**

Muhammad Imran Qadeer, Ali Amar, Yung-Yu Huang, Eli Min, Hanga Galfalvy, Shahida Hasnain and J. John Mann

**Supplementary Table S1: Basic characteristics of subjects included in this study**

| **Characteristics** | **Frequency in prison inmates (n = 370)** | **Frequency in normal controls (n = 359)** | ***p*-value** |
| --- | --- | --- | --- |
| Age (years)^1^ | 36.4±11.8 | 35.2±10.7 | 0.177 |
| **District of Punjab** | **n = 370*** | **n = 354*** |  |
| Sargodha | 181 (48.8%) | 186 (52.5%) | 0.456 |
| Toba tek singh | 99 (26.7%) | 81 (22.9%) |  |
| Mianwali | 90 (24.3%) | 87 (24.6%) |  |
| **Socio-economic status** | **n = 362*** | **n = 352*** |  |
| Poor | 80 (22.1%) | 79 (22.4%) | 0.677 |
| Middle class | 261 (72.1%) | 247 (70.2%) |  |
| Affluent | 21 (5.8%) | 26 (7.4%) |  |
| **Educational level** | **n = 366*** | **n = 352*** |  |
| Illiterate | 137 (37.4%) | 115 (32.7%) | 0.296 |
| High School education | 215 (58.7%) | 218 (61.9%) |  |
| Graduate and above | 14 (3.8%) | 19 (5.4%) |  |
| **Marital status** | **n = 366*** |  |  |
| Unmarried | 150 (40.9%) | - |  |
| Single marriage | 205 (56%) | - |  |
| Complicated | 11 (03%) | - |  |
| **Type of sentenced prisoner^2^** | **n = 365*** |  |  |
| Under trial/alleged | 87 (23.8%) | - |  |
| Convicted | 25 (6.8%) | - |  |
| Condemned | 253 (69.2%) | - |  |
| **Age at time of committing murder^a^** | 30.4±11.1 | - |  |
| **Motive of murder** | **n = 292*** |  |  |
| Honor | 101 (34.6%) | - |  |
| Property | 56 (19.2%) | - |  |
| Revenge | 134 (45.9%) | - |  |
| Without any reason | 01 (0.3%) | - |  |
| **Murdered by** | **n = 297*** |  |  |
| Gun shots | 243 (81.8%) | - |  |
| Stabbing/execution | 36 (12.1%) | - |  |
| Beating with solid objects | 11 (3.7%) | - |  |
| Strangling | 07 (2.4%) | - |  |
| **Relationship with murdered victims** | **n = 297*** |  |  |
| Blood relative | 117 (39.4%) | - |  |
| From in laws | 22 (7.4%) | - |  |
| Rival | 71 (23.9%) | - |  |
| Stranger | 87 (29.3%) | - |  |
| **Family history of murder** |  |  |  |
| Yes | 72 (24.9%) | - |  |
| No | 216 (75.1%) | - |  |
| **Anyone got murdered in the family** | **n = 288*** |  |  |
| Yes | 70 (24.1%) | - |  |
| No | 221 (75.9%) | - |  |
| **Self-reported aggression** | **n = 358*** |  |  |
| Yes | 242 (67.4%) | - |  |
| No | 116 (32.6%) | - |  |
| **Provoked by physical abuse** | **n = 323*** |  |  |
| Yes | 199 (61.4%) | - |  |
| No | 124 (38.6%) | - |  |
| **Provoked by verbal abuse** | **n = 322*** |  |  |
| Yes | 204 (63.2%) | - |  |
| No | 118 (36.8%) | - |  |
| **Parental marital history** | **n = 365*** |  |  |
| Single marriage | 301 (82.2%) | - |  |
| Complicated | 64 (17.8%) | - |  |
| **Step relations** | **n = 362*** |  |  |
| Yes | 86 (23.7%) | - |  |
| No | 276 (76.3%) | - |  |
| **History of parental aggression** | **n = 360*** |  |  |
| Yes | 185 (51.5%) | - |  |
| No | 175 (48.5%) | - |  |
| **Childhood history of abuse** | **n = 361*** |  |  |
| Yes | 230 (63.8%) | - |  |
| No | 131 (36.2%) | - |  |
| **Any minor psychiatric problem**^3^ | **n = 322*** |  |  |
| Yes | 17 (5.3%) | - |  |
| No | 305 (94.7%) | - |  |
| **Substance use disorder** | **n = 347*** |  |  |
| Yes | 69 (19.8%) | - |  |
| No | 278 (80.2%) | - |  |

^1^Data is mean ± SD

^2^The sentenced prisoners guilty of homicidal behavior were classified into three sub-groups/statuses as per prison staff. The under-trial/alleged status refers to murderers who were sentenced for murder but appealed in another higher court of law or to the President of Pakistan for reduced sentences. The convicted status here refers to murderers sentenced to a life time imprisonment instead of death sentence (*e.g.* for young individuals). Whereas, condemned status here describes murderers sentenced to receive a death punishment.

^3^Any minor psychiatric problem here refers to minor somatic problems involving non-psychotic psychiatric problems including insomnia, fatigue, irritability, forgetfulness and difficulty in concentrating*.* It is to be noted that prison inmates with a diagnosis or family history of any DSM-IV psychiatric disorder were excluded from the study.

*The total number differs from variable to variable because information was either not available or not provided for that variable.

**Supplementary Table S2: Basic information and HWE analysis for studied polymorphisms of serotonergic system genes**

| **Polymorphism** | **Gene** | **Type/rs number** | **Region** | **Allele** | **MAF** | | ***p*-value for HWE^1^** | **Samples genotyped (%)** |
| --- | --- | --- | --- | --- | --- | --- | --- | --- |
|  |  |  |  |  | **Cases**  **(n = 370)** | **Controls**  **(n = 359)** |  |  |
| HTTLPR (44 bp VNTR polymorphism) | *5HTT* | Insertion/deletion | Promoter | L | 0.46 | 0.39 | 0.99 | 97 |
| T/C variant in HTTLPR (tri-allelic polymorphism) | *5HTT* | rs25531 | Promoter | L_A_ | 0.34 | 0.28 | 0.80 | 97 |
| G/T SNP | *5HTT* | rs1042173 | 3ʹ UTR | T | 0.45 | 0.43 | 0.096 | 93 |
| STin2 (17 bp VNTR polymorphism) | *5HTT* | Insertion/deletion | Intron 02 | 10R | 0.33 | 0.30 | 0.90 | 98 |
| C/T SNP | *HTR2A* | rs6311 | Promoter | T | 0.41 | 0.42 | 0.59 | 99 |
| C/T SNP | *HTR2B* | rs17440378 | Intron 02 | T | 0.16 | 0.13 | 0.33 | 96 |

HWE, Hardy–Weinberg equilibrium; MAF, minor allele frequencies; SNP, single-nucleotide polymorphism; UTR, untranslated region; VNTR, variable number of tandem repeats

^1^HWE *p*-value in the control group.

**
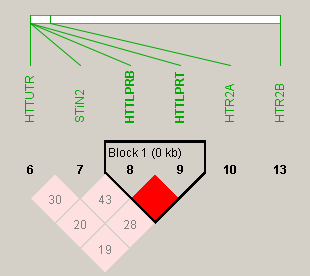
**

**Supplementary Figure S1: Linkage disequilibrium (LD) pattern between serotonergic polymorphisms analyzed**. Lewontin’s D՛ (%) values of linkage disequilibrium between each serotonergic polymorphic pair, as determined by Haploview program, revealed no significant LD except for bi-allelic (HTTLPRB) and tri-allelic (HTTLPRT) HTTLPR polymorphisms in *5HTT* gene (Block 1). Higher values and darker colors indicate stronger LD between loci pairs.

**Supplementary Table S3: Distribution of combined genotype and haplotype frequencies of the studied polymorphisms in serotonergic system genes (*5HTT*, *HTR2A* and *HTR2B*) and their association with history of homicide**

| **Combined genotypes** | | | | | | | **Combined genotype/haplotype frequencies^1^** | | **OR (95% CI)** | ***p*-value** |
| --- | --- | --- | --- | --- | --- | --- | --- | --- | --- | --- |
| ***5HTT*  3ʹ UTR (rs1042173)** | | ***5HTT* STin2** | **HTTLPRB** | **HTTLPRT** | ***HTR2A* rs6311** | ***HTR2B* rs17440378** | **Cases** | **Controls** |  |  |
|  | **Combined genotype analysis of all the studied polymorphisms in serotonergic system genes^2^** | | | | | | | | | |
| G | | 12R | S | S/L_G_ | C | C | 0.156 | 0.185 | Referent | - |
| G | | 12R | S | S/L_G_ | T | C | 0.103 | 0.134 | 0.91 (0.55-1.51) | 0.720 |
| T | | 12R | S | S/L_G_ | C | C | 0.065 | 0.073 | 1.08 (0.56-2.07) | 0.820 |
| T | | 12R | S | S/L_G_ | T | C | 0.050 | 0.062 | 0.87 (0.46-1.63) | 0.660 |
| T | | 10R | L | L_A_ | C | C | 0.057 | 0.047 | 1.50 (0.74-3.20) | 0.260 |
|  | Global combined genotypes *p*-value | | | | | | | | | 0.035 |
|  | **Haplotype analysis of bi-allelic and tri-allelic HTTLPR polymorphisms according to LD block structure^2^** | | | | | | | | | |
| - | | - | S | S/L_G_ | - |  | 0.544 | 0.608 | Referent | - |
| - | | - | L | L_A_ | - |  | 0.344 | 0.284 | **1.35 (1.07-1.71)** | **0.011** |
| - | | - | L | S/L_G_ | - |  | 0.112 | 0.108 | 1.15 (0.83-1.60) | 0.400 |
|  | Global haplotype *p*-value | | | | | | | | | 0.036 |

OR – odds ratio; 95% CI – 95% confidence interval.

^1^Combined genotypes/haplotypes with a frequency >5% were analyzed.

^2^Bonferroni correction for multiple testing was applied and a *p*-value of < 0.01 and < 0.016 was considered significant for combined genotype (considering all the serotonergic polymorphisms in both *5HTT* and *HTR2A* genes) and haplotype (bi-allelic and tri-allelic HTTLPR polymorphisms in *5HTT* gene only) analyses, respectively. Statistically significant *p*-values and associated OR values are highlighted in bold.

**Supplementary Table S4 A: Association of *5HTT* STin2 polymorphism with self-reported aggression and histories**

| **Response to measure** | ***5HTT* STin2 genotypes (recessive model)** | | **OR (95% CI)** | ***p*-value** |
| --- | --- | --- | --- | --- |
|  | **12R/12R-12R/10R, n (%)** | **10R/10R, n (%)** |  |  |
| **Self-reported aggression (Lifetime)** | | | | |
| Yes | 207 (67.2%) | 30 (76.9%) | 1.63 (0.74-3.56) | 0.223 |
| No | 101 (32.8%) | 09 (23.1%) |  |  |
| **Provoked by verbal abuse** | | | | |
| Yes | 179 (63.9%) | 22 (68.8%) | 1.24 (0.57-2.73) | 0.590 |
| No | 101 (36.1%) | 10 (31.3%) |  |  |
| **Provoked by physical abuse** | | | | |
| Yes | 176 (62.6%) | 20 (62.5%) | 0.99 (0.47-2.12) | 0.988 |
| No | 105 (37.4%) | 12 (37.5%) |  |  |
| **Childhood history of abuse** | | | | |
| Yes | 198 (63.7%) | 26 (66.7%) | 1.14 (0.56-2.31) | 0.713 |
| No | 113 (36.3%) | 13 (33.3%) |  |  |
| **History of parental aggression towards future aggressor (murderer)** | | | | |
| Yes | 157 (50.5%) | 26 (66.7%) | 1.96 (0.97-3.96) | 0.060 |
| No | 154 (49.5%) | 13 (33.3%) |  |  |
| **Parental marital problems (including divorced and separated with step parents)** | | | | |
| Yes | 81 (25.6%) | 10 (25.6%) | 1.00 (0.47-2.14) | 0.999 |
| No | 235 (74.4%) | 29 (74.4%) |  |  |
| **Psychiatric illness** | | | | |
| Yes | 13 (4.7%) | 03 (8.6%) | 1.90 (0.52-7.04) | 0.335 |
| No | 264 (95.3%) | 32 (91.4%) |  |  |
| **Substance use disorder** | | | | |
| Yes | 60 (20.1%) | 05 (13.2%) | 0.60 (0.23-1.61) | 0.310 |
| No | 238 (79.9%) | 33 (86.8%) |  |  |

OR, odds ratio; CI, confidence interval. Boldface indicates *p* < 0.05 was considered as statistically significant.

**Supplementary Table S4 B: Association of *5HTT* 3ʹ UTR (rs1042173) polymorphism with self-reported aggression and histories**

| **Response to measure** | ***5HTT* 3ʹ UTR genotypes (recessive model)** | | **OR (95% CI)** | ***p*-value** |
| --- | --- | --- | --- | --- |
|  | **G/G-G/T, n (%)** | **T/T, n (%)** |  |  |
| **Self-reported aggression (Lifetime)** | | | | |
| Yes | 165 (66.3%) | 59 (72.0%) | 1.31 (0.75-2.26) | 0.340 |
| No | 84 (33.7%) | 23 (28.0%) |  |  |
| **Provoked by verbal abuse** | | | | |
| Yes | 141 (63.2%) | 48 (64.9%) | 1.07 (0.62-1.86) | 0.800 |
| No | 82 (36.8%) | 26 (35.1%) |  |  |
| **Provoked by physical abuse** | | | | |
| Yes | 137 (61.2%) | 46 (62.2%) | 1.04 (0.61-1.79) | 0.878 |
| No | 87 (38.8%) | 28 (37.8%) |  |  |
| **Childhood history of abuse** | | | | |
| Yes | 168 (66.7%) | 45 (54.9%) | 0.61 (0.37-1.01) | 0.055 |
| No | 84 (33.3%) | 37 (45.1%) |  |  |
| **History of parental aggression towards future aggressor (murderer)** | | | | |
| Yes | 122 (48.8%) | 46 (56.1%) | 1.34 (0.81-2.21) | 0.252 |
| No | 128 (51.2%) | 36 (43.9%) |  |  |
| **Parental marital problems (including divorced and separated with step parents)** | | | | |
| Yes | 63 (24.7%) | 22 (26.5%) | 1.10 (0.63-1.93) | 0.743 |
| No | 192 (75.3%) | 61 (73.5%) |  |  |
| **Psychiatric illness** | | | | |
| Yes | 13 (5.8%) | 03 (4.1%) | 0.70 (0.19-2.51) | 0.580 |
| No | 211 (94.2%) | 70 (95.9%) |  |  |
| **Substance use disorder** | | | | |
| Yes | 50 (20.7%) | 12 (15.2%) | 0.68 (0.34-1.36) | 0.280 |
| No | 191 (79.3%) | 67 (84.8%) |  |  |

OR, odds ratio; CI, confidence interval. Boldface indicates *p* < 0.05 was considered as statistically significant.

**Supplementary Table S4 C: Association of *HTR2A* rs6311 polymorphism with self-reported aggression and histories**

| **Response to measure** | ***HTR2A* genotypes (recessive model)** | | **OR (95% CI)** | ***p*-value** |
| --- | --- | --- | --- | --- |
|  | **C/C-C/T, n (%)** | **T/T, n (%)** |  |  |
| **Self-reported aggression (Lifetime)** | | | | |
| Yes | 202 (68.2%) | 37 (67.3%) | 0.96 (0.52-1.77) | 0.887 |
| No | 94 (31.8%) | 18 (32.7%) |  |  |
| **Provoked by verbal abuse** | | | | |
| Yes | 171 (63.8%) | 31 (62.0%) | 0.92 (0.50-1.73) | 0.808 |
| No | 97 (36.2%) | 19 (38.0%) |  |  |
| **Provoked by physical abuse** | | | | |
| Yes | 170 (63.2%) | 28 (56.0%) | 0.74 (0.40-1.37) | 0.337 |
| No | 99 (36.8%) | 22 (44.0%) |  |  |
| **Childhood history of abuse** | | | | |
| Yes | 190 (63.5%) | 35 (63.6%) | 1.00 (0.55-1.83) | 0.990 |
| No | 109 (36.5%) | 20 (36.4%) |  |  |
| **History of parental aggression towards future aggressor (murderer)** | | | | |
| Yes | 156 (52.7%) | 26 (45.6%) | 0.75 (0.43-1.33) | 0.328 |
| No | 140 (47.3%) | 31 (54.4%) |  |  |
| **Parental marital problems (including divorced and separated with step parents)** | | | | |
| Yes | 79 (26.2%) | 12 (21.1%) | 0.75 (3.80-1.50) | 0.417 |
| No | 223 (73.8%) | 45 (78.9%) |  |  |
| **Psychiatric illness** | | | | |
| Yes | 14 (5.3%) | 03 (5.9%) | 1.12 (0.31-4.05) | 0.862 |
| No | 251 (94.7%) | 48 (94.1%) |  |  |
| **Substance use disorder** | | | | |
| Yes | 60 (20.9%) | 07 (12.7%) | 0.55 (0.24-1.28) | 0.167 |
| No | 227 (79.1%) | 48 (87.3%) |  |  |

OR, odds ratio; CI, confidence interval. Boldface indicates *p* < 0.05 was considered as statistically significant.

**Supplementary Table S4 D: Association of tri-allelic rs25531 in HTTLPR polymorphism with self-reported aggression and histories**

| **Genotypes of rs25531 in HTTLPR** | **Response to measure** | | **OR (95% CI)** | ***p*-value** |
| --- | --- | --- | --- | --- |
|  | **Yes, n (%)** | **No, n (%)** |  |  |
| **Self-reported aggression (Lifetime)** | | | | |
| Low (S/S, S/L_G_, L_G_/L_G_) | 103 (43.1%) | 50 (44.6%) | Referent | - |
| Intermediate (L_A_/S, L_A_/L_G_) | 103 (43.1%) | 51 (45.5%) | 0.98 (0.61-1.57) | 0.935 |
| High (L_A_/L_A_) | 33 (13.8%) | 11 (9.8%) | 1.46 (0.68-3.12) | 0.333 |
| **Provoked by verbal abuse** | | | | |
| Low (S/S, S/L_G_, L_G_/L_G_) | 91 (44.6%) | 50 (43.9%) | Referent | - |
| Intermediate (L_A_/S, L_A_/L_G_) | 83 (40.7%) | 53 (40.7%) | 0.86 (0.53-1.40) | 0.546 |
| High (L_A_/L_A_) | 30 (14.7%) | 11 (9.6%) | 1.50 (0.69-3.24) | 0.305 |
| **Provoked by physical abuse** | | | | |
| Low (S/S, S/L_G_, L_G_/L_G_) | 89 (44.7%) | 53 (44.2%) | Referent | - |
| Intermediate (L_A_/S, L_A_/L_G_) | 81 (40.7%) | 55 (45.8%) | 0.88 (0.54-1.42) | 0.594 |
| High (L_A_/L_A_) | 29 (14.6%) | 12 (10.0%) | 1.44 (0.68-3.06) | 0.344 |
| **Childhood history of abuse** | | | | |
| Low (S/S, S/L_G_, L_G_/L_G_) | 105 (46.1%) | 47 (37.6%) | Referent | - |
| Intermediate (L_A_/S, L_A_/L_G_) | 92 (40.4%) | 65 (52.0%) | 0.63 (0.40-1.01) | 0.056 |
| High (L_A_/L_A_) | 31 (13.6%) | 13 (10.4%) | 1.07 (0.51-2.22) | 0.862 |
| **History of parental aggression towards future aggressor (murderer)** | | | | |
| Low (S/S, S/L_G_, L_G_/L_G_) | 81 (44.8%) | 72 (41.9%) | Referent | - |
| Intermediate (L_A_/S, L_A_/L_G_) | 78 (43.1%) | 78 (45.3%) | 0.90 (0.57-1.39) | 0.605 |
| High (L_A_/L_A_) | 22 (12.2%) | 22 (12.8%) | 0.90 (0.45-1.74) | 0.731 |
| **Parental marital problems (including divorced and separated with step parents)** | | | | |
| Low (S/S, S/L_G_, L_G_/L_G_) | 39 (42.4%) | 117 (44.0%) | Referent | - |
| Intermediate (L_A_/S, L_A_/L_G_) | 45 (48.9%) | 113 (42.5%) | 1.20 (0.72-1.97) | 0.486 |
| High (L_A_/L_A_) | 08 (8.7%) | 36 (13.5%) | 0.67 (0.29-1.56) | 0.348 |
| **Psychiatric illness** | | | | |
| Low (S/S, S/L_G_, L_G_/L_G_) | 11 (73.3%) | 128 (42.7%) | Referent | - |
| Intermediate (L_A_/S, L_A_/L_G_) | 03 (20.0%) | 132 (44.0%) | **0.26 (0.07-0.97)** | **0.045** |
| High (L_A_/L_A_) | 01 (6.7%) | 40 (13.3%) | 0.29 (0.03-2.32) | 0.244 |
| **Substance use disorder** | | | | |
| Low (S/S, S/L_G_, L_G_/L_G_) | 36 (52.9%) | 111 (41.0%) | Referent | - |
| Intermediate (L_A_/S, L_A_/L_G_) | 24 (35.3%) | 127 (46.9%) | 0.58 (0.33-1.04) | 0.066 |
| High (L_A_/L_A_) | 08 (11.8%) | 33 (12.2%) | 0.75 (0.32-1.77) | 0.507 |

OR, odds ratio; CI, confidence interval. Boldface indicates *p* < 0.05 was considered as statistically significant.

**Supplementary Table S4 E: Association of *HTR2B* rs17440378 polymorphism with self-reported aggression and histories**

| **Response to measure** | ***HTR2B* genotypes (dominant model)** | | **OR (95% CI)** | ***p*-value** |
| --- | --- | --- | --- | --- |
|  | **C/C, n (%)** | **C/T-T/T, n (%)** |  |  |
| **Self-reported aggression (Lifetime)** | | | | |
| Yes | 171 (69%) | 69 (67%) | 0.91 (0.56-1.49) | 0.82 |
| No | 77 (31%) | 34 (33%) |  |  |
| **Provoked by verbal abuse** | | | | |
| Yes | 149 (65.6%) | 55 (61.1%) | 0.82 (0.50-1.36) | 0.53 |
| No | 78 (34.4%) | 35 (38.9%) |  |  |
| **Provoked by physical abuse** | | | | |
| Yes | 145 (63.6%) | 54 (60%) | 0.86 (0.52-1.42) | 0.64 |
| No | 83 (36.4%) | 36 (40%) |  |  |
| **Childhood history of abuse** | | | | |
| Yes | 160 (64%) | 67 (65%) | 1.05 (0.65-1.69) | 1.00 |
| No | 90 (36%) | 36 (35%) |  |  |
| **History of parental aggression towards future aggressor (murderer)** | | | | |
| Yes | 128 (51.2%) | 54 (52.4%) | 1.05 (0.66-1.66) | 0.92 |
| No | 122 (48.8%) | 49 (47.6%) |  |  |
| **Parental marital problems (including divorced and separated with step parents)** | | | | |
| Yes | 56 (22.1%) | 35 (33.3%) | **1.76 (1.06-2.91)** | **0.037** |
| No | 197 (77.9%) | 70 (66.7%) |  |  |
| **Psychiatric illness** | | | | |
| Yes | 08 (3.6%) | 08 (8.9%) | 2.64 (0.96-7.28) | 0.08 |
| No | 217 (96.4%) | 82 (91.1%) |  |  |
| **Substance use disorder** | | | | |
| Yes | 49 (20.2%) | 19 (19.6%) | 0.96 (0.53-1.74) | 1.00 |
| No | 194 (79.8%) | 78 (80.4%) |  |  |

OR, odds ratio; CI, confidence interval. Boldface indicates *p* < 0.05 was considered as statistically significant.

**Supplementary Table S5 A: Association of 5*HTT* STin2 polymorphism with different environmental parameters including demographics**

| **Measure** | **5*HTT* STin2 (Recessive model)** | | **OR (95% CI)** | ***p*-value** |
| --- | --- | --- | --- | --- |
|  | **12R/12R-12R/10R, n (%)** | **10R/10R, n (%)** |  |  |
| **Age at time of enrollment** | | | | |
| <25y (Adolescents) | 43 (13.4%) | 4 (10.3%) | 0.73 (0.24-2.17) | 0.632 |
| 25y or more (Adults) | 277 (86.6%) | 35 (89.7%) |  |  |
| **Age at time of committing murder** | | | | |
| <25y (Adolescents) | 117 (36.6%) | 11 (28.2%) | 0.68 (0.32-1.41) | 0.303 |
| 25y or more (Adults) | 203 (63.4%) | 28 (71.8%) |  |  |
| **Region** | | | | |
| Southern Punjab | 91 (28.4%) | 8 (20.5%) | 0.64 (0.28-1.46) | 0.296 |
| Northern + Central Punjab | 229 (71.6%) | 31 (79.5%) |  |  |
| **Socio-economic status** | | | | |
| Poor | 70 (22.2%) | 7 (18.9%) | 0.81 (0.34-1.98) | 0.64 |
| Middle class & affluent | 245 (77.8%) | 30 (81.1%) |  |  |
| **Educational status** | | | | |
| Less than high school | 120 (38%) | 14 (35.9%) | 0.91 (0.45-1.82) | 0.806 |
| High school or more | 196 (62%) | 25 (64.1%) |  |  |
| **Caste (ethnic groups)** | | | | |
| Arian | 31 (9.7%) | 4 (10.3%) | - | 0.274 |
| Jutt | 129 (40.4%) | 20 (51.3%) |  |  |
| Pathan | 59 (18.5%) | 2 (5.1%) |  |  |
| Rajpot | 68 (21.3%) | 10 (25.6%) |  |  |
| Syed | 32 (10%) | 3 (7.7%) |  |  |
| **Marital status** | | | | |
| Married | 191 (60.4%) | 21 (53.8%) | 0.76 (0.39-1.49) | 0.427 |
| Unmarried | 125 (39.6%) | 18 (46.2%) |  |  |

OR, odds ratio; CI, confidence interval

Boldface indicates *p* < 0.05 was considered as statistically significant.

**Supplementary Table S5 B: Association of 5*HTT* 3’UTR (rs1042173) polymorphism with different environmental parameters including demographics**

| **Measure** | **5*HTT* 3’UTR (Recessive model)** | | **OR (95% CI)** | ***p*-value** |
| --- | --- | --- | --- | --- |
|  | **G/G-G/T, n (%)** | **T/T, n (%)** |  |  |
| **Age at time of enrollment** | | | | |
| <25y (Adolescents) | 37 (14.3%) | 10 (11.9%) | 0.80 (0.38-1.70) | 0.571 |
| 25y or more (Adults) | 221 (85.7%) | 74 (88.1%) |  |  |
| **Age at time of committing murder** | | | | |
| <25y (Adolescents) | 95 (36.8%) | 30 (35.7%) | 0.95 (0.57-1.59) | 0.862 |
| 25y or more (Adults) | 163 (63.2%) | 54 (64.3%) |  |  |
| **Region** | | | | |
| Southern Punjab | 67 (26%) | 28 (33.3%) | 1.42 (0.83-2.42) | 0.190 |
| Northern + Central Punjab | 191 (74%) | 56 (66.7%) |  |  |
| **Socio-economic status** | | | | |
| Poor | 62 (24.6%) | 14 (17.1%) | 0.63 (0.33-1.19) | 0.157 |
| Middle class & affluent | 190 (75.4%) | 68 (82.9%) |  |  |
| **Educational status** | | | | |
| Less than high school | 99 (38.8%) | 31 (37.3%) | 0.93 (0.56-1.56) | 0.806 |
| High school or more | 156 (61.2%) | 52 (62.7%) |  |  |
| **Caste (ethnic groups)** | | | | |
| Arian | 21 (8.2%) | 11 (13.1%) | - | 0.448 |
| Jutt | 103 (40.1%) | 38 (45.2%) |  |  |
| Pathan | 46 (17.9%) | 14 (16.7%) |  |  |
| Rajpot | 60 (23.3%) | 14 (16.7%) |  |  |
| Syed | 27 (10.5%) | 7 (8.3%) |  |  |
| **Marital status** | | | | |
| Married | 150 (58.8%) | 50 (60.2%) | 1.06 (0.63-1.75) | 0.823 |
| Unmarried | 105 (41.2%) | 33 (39.8%) |  |  |

OR, odds ratio; CI, confidence interval

Boldface indicates *p* < 0.05 was considered as statistically significant.

**Supplementary Table S5 C: Association of HTTLPR VNTR polymorphism in *5HTT* gene with different environmental parameters including demographics**

| **Measure** | **HTTLPR (Recessive model)** | | **OR (95% CI)** | ***p*-value** |
| --- | --- | --- | --- | --- |
|  | **S/S-S/L, n (%)** | **L/L, n (%)** |  |  |
| **Age at time of enrollment** | | | | |
| <25y (Adolescents) | 36 (12.7%) | 14 (17.7%) | 1.47 (0.75-2.90) | 0.254 |
| 25y or more (Adults) | 247 (87.3%) | 65 (82.3%) |  |  |
| **Age at time of committing murder** | | | | |
| <25y (Adolescents) | 98 (34.6%) | 33 (41.8%) | 1.35 (0.81-2.25) | 0.243 |
| 25y or more (Adults) | 185 (65.4%) | 46 (58.2%) |  |  |
| **Region** | | | | |
| Southern Punjab | 78 (27.6%) | 21 (26.6%) | 0.95 (0.54-1.67) | 0.862 |
| Northern + Central Punjab | 205 (72.4%) | 58 (73.4%) |  |  |
| **Socio-economic status** | | | | |
| Poor | 64 (23%) | 14 (18.2%) | 0.74 (0.39-1.41) | 0.365 |
| Middle class & affluent | 214 (77%) | 63 (81.8%) |  |  |
| **Educational status** | | | | |
| Less than high school | 110 (39%) | 26 (33.8%) | 0.79 (0.46-1.35) | 0.399 |
| High school or more | 172 (61%) | 51 (66.2%) |  |  |
| **Caste (ethnic groups)** | | | | |
| Arian | 30 (10.6%) | 6 (7.7%) | - | 0.491 |
| Jutt | 123 (43.5%) | 28 (35.9%) |  |  |
| Pathan | 45 (15.9%) | 16 (20.5%) |  |  |
| Rajpot | 61 (21.6%) | 18 (23.1%) |  |  |
| Syed | 24 (8.5%) | 10 (12.8%) |  |  |
| **Marital status** | | | | |
| Married | 173 (61.6%) | 40 (51.9%) | 0.67 (0.40-1.12) | 0.127 |
| Unmarried | 108 (38.4%) | 37 (48.1%) |  |  |

OR, odds ratio; CI, confidence interval

Boldface indicates *p* < 0.05 was considered as statistically significant.

**Supplementary Table S5 D: Association of tri-allelic rs25531 in HTTLPR polymorphism with different environmental parameters including demographics**

| **Measure** | **rs25531 in HTTLPR (Recessive model)** | | **OR (95% CI)** | ***p*-value** |
| --- | --- | --- | --- | --- |
|  | **Relatively low expression genotypes (S/S, S/L_G_, L_G_/L_G_, L_A_/S, L_A_/L_G_)** **, n (%)** | **High expression genotype (L_A_/L_A_)** **, n (%)** |  |  |
| **Age at time of enrollment** | | | | |
| <25y (Adolescents) | 40 (12.6%) | 10 (22.7%) | 2.04 (0.93-4.45) | 0.067 |
| 25y or more (Adults) | 278 (87.4%) | 34 (77.3%) |  |  |
| **Age at time of committing murder** | | | | |
| <25y (Adolescents) | 112 (35.2%) | 19 (43.2%) | 1.39 (0.73-2.64) | 0.303 |
| 25y or more (Adults) | 206 (64.8%) | 25 (56.8%) |  |  |
| **Region** | | | | |
| Southern Punjab | 87 (27.4%) | 12 (27.3%) | 0.99 (0.49-2.02) | 1.00 |
| Northern + Central Punjab | 231 (72.6%) | 32 (72.7%) |  |  |
| **Socio-economic status** | | | | |
| Poor | 69 (22.2%) | 9 (20.5%) | 0.90 (0.41-1.96) | 0.791 |
| Middle class & affluent | 242 (77.8%) | 35 (79.5%) |  |  |
| **Educational status** | | | | |
| Less than high school | 120 (38.1%) | 16 (36.4%) | 0.92 (0.48-1.78) | 0.823 |
| High school or more | 195 (61.9%) | 28 (63.6%) |  |  |
| **Caste (ethnic groups)** | | | | |
| Arian | 34 (10.7%) | 2 (4.5%) | - | 0.214 |
| Jutt | 131 (41.3%) | 20 (45.5%) |  |  |
| Pathan | 52 (16.4%) | 9 (20.5%) |  |  |
| Rajpot | 73 (23%) | 6 (13.6%) |  |  |
| Syed | 27 (8.5%) | 7 (15.9%) |  |  |
| **Marital status** | | | | |
| Married | 188 (59.9%) | 25 (56.8%) | 0.88 (0.46-1.66) | 0.698 |
| Unmarried | 126 (40.1%) | 19 (43.2%) |  |  |

OR, odds ratio; CI, confidence interval

Boldface indicates *p* < 0.05 was considered as statistically significant.

**Supplementary Table S5 E: Association of *HTR2A* rs6311 polymorphism with different environmental parameters including demographics**

| **Measure** | ***HTR2A* rs6311 (Recessive model)** | | **OR (95% CI)** | ***p*-value** |
| --- | --- | --- | --- | --- |
|  | **C/C-C/T, n (%)** | **T/T, n (%)** |  |  |
| **Age at time of enrollment** | | | | |
| <25y (Adolescents) | 44 (14.4%) | 5 (8.8%) | 0.57 (0.21-1.51) | 0.256 |
| 25y or more (Adults) | 262 (85.6%) | 52 (91.2%) |  |  |
| **Age at time of committing murder** | | | | |
| <25y (Adolescents) | 105 (34.3%) | 22 (38.6%) | 1.20 (0.67-2.15) | 0.532 |
| 25y or more (Adults) | 201 (65.7%) | 35 (61.4%) |  |  |
| **Region** | | | | |
| Southern Punjab | 85 (27.8%) | 12 (21.1%) | 0.69 (0.34-1.37) | 0.292 |
| Northern + Central Punjab | 221 (72.2%) | 45 (78.9%) |  |  |
| **Socio-economic status** | | | | |
| Poor | 66 (22.1%) | 11 (19.6%) | 0.86 (0.42-1.76) | 0.689 |
| Middle class & affluent | 233 (77.9%) | 45 (80.4%) |  |  |
| **Educational status** | | | | |
| Less than high school | 117 (38.6%) | 17 (30.4%) | 0.69 (0.37-1.28) | 0.240 |
| High school or more | 186 (61.4%) | 39 (69.6%) |  |  |
| **Caste (ethnic groups)** | | | | |
| Arian | 29 (9.5%) | 6 (10.5%) | - | 0.990 |
| Jutt | 129 (42.3%) | 22 (38.6%) |  |  |
| Pathan | 52 (17%) | 10 (17.5%) |  |  |
| Rajpot | 66 (21.6%) | 13 (22.8%) |  |  |
| Syed | 29 (9.5%) | 6 (10.5%) |  |  |
| **Marital status** | | | | |
| Married | 177 (58.6%) | 36 (63.2%) | 1.21 (0.67-2.17) | 0.521 |
| Unmarried | 125 (41.4%) | 21 (36.8%) |  |  |

OR, odds ratio; CI, confidence interval

Boldface indicates *p* < 0.05 was considered as statistically significant.

**Supplementary Table S5 F: Association of *HTR2B* rs17440378 polymorphism with different environmental parameters including demographics**

| **Measure** | ***HTR2B rs17440378* (Dominant model)** | | **OR (95% CI)** | ***p*-value** |
| --- | --- | --- | --- | --- |
|  | **C/C, n (%)** | **C/T-T/T, n (%)** |  |  |
| **Age at time of enrollment** | | | | |
| <25y (Adolescents) | 39 (15.2%) | 11 (10.5%) | 0.65 (0.32-1.33) | 0.31 |
| 25y or more (Adults) | 218 (84.8%) | 94 (89.5%) |  |  |
| **Age at time of committing murder** | | | | |
| <25y (Adolescents) | 98 (38.1%) | 31 (29.5%) | 0.67 (0.42-1.11) | 0.15 |
| 25y or more (Adults) | 159 (61.9%) | 74 (70.5%) |  |  |
| **Region** | | | | |
| Southern Punjab | 69 (36.8%) | 29 (27.6%) | 1.04 (0.62-1.73) | 1.00 |
| Northern + Central Punjab | 188 (73.2 %) | 76 (72.4%) |  |  |
| **Socio-economic status** | | | | |
| Poor | 57 (22.8%) | 19 (18.3%) | 1.13 (0.62-2.05) | 0.81 |
| Middle class & affluent | 193 (77.2%) | 85 (81.7%) |  |  |
| **Educational status** | | | | |
| Less than high school | 96 (37.9%) | 37 (35.2%) | 0.89 (0.55-1.43) | 0.72 |
| High school or more | 157 (62.1%) | 68 (64.8%) |  |  |
| **Caste (ethnic groups)** | | | | |
| Arian | 24 (9.3%) | 12 (11.5%) | - | 0.71 |
| Jutt | 113 (44%) | 38 (36.5%) |  |  |
| Pathan | 43 (16.7%) | 18 (17.3%) |  |  |
| Rajpot | 52 (20.2%) | 26 (25%) |  |  |
| Syed | 25 (9.7%) | 10 (9.6%) |  |  |
| **Marital status** | | | | |
| Married | 104 (41.1%) | 42 (40%) | 0.96 (0.60-1.52) | 0.92 |
| Unmarried | 149 (58.9%) | 63 (60%) |  |  |

OR, odds ratio; CI, confidence interval

Boldface indicates *p* < 0.05 was considered as statistically significant.


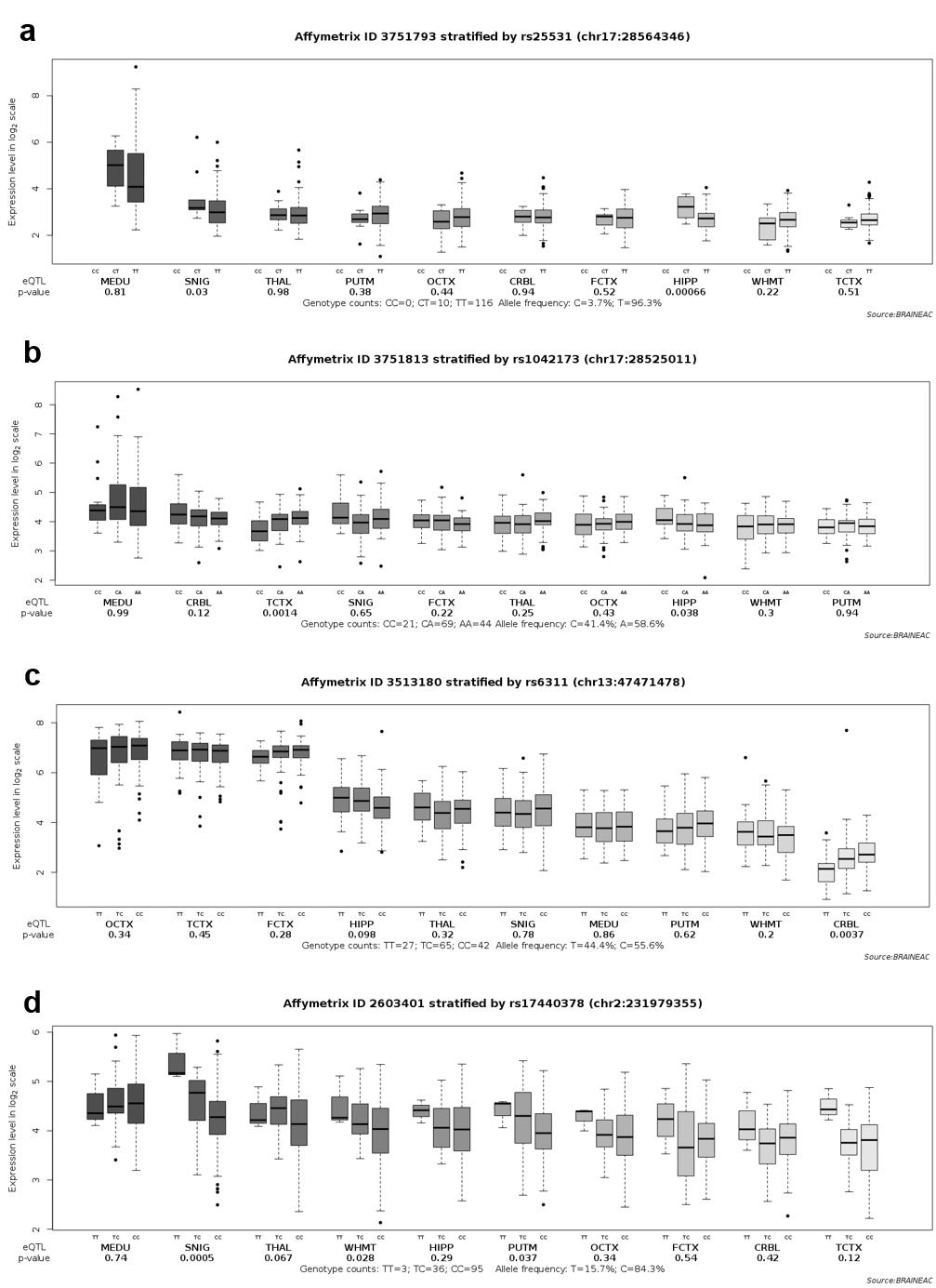


**Supplementary Figure S2: Serotonergic system polymorphisms modulate expression of *SLC6A4,* *HTR2A* and *HTR2B* genes in different normal human brain regions in Braineac. (a)** rs25531 in 5-HTTLPR is a significant eQTL of *SLC6A4* expression in hippocampus (*p* = 6.6 x 10^-4^), **(b)** A allele of rs1042173 polymorphism is associated with more pronounced expression of *SLC6A4* gene in temporal cortex region (*p* = 1.4 x 10^-3^), **(c)** relatively low expression of *HTR2A* receptor is apparent for TT genotype (vs TC and CC genotypes) of rs6311 polymorphism in cerebellar cortex (*p* = 3.7 x 10^-3^), and **(d)** significant eQTL effects were identified for *HTR2B* rs17440378 polymorphism in substantia nigra region of the normal human brain (*p* = 5.0 x 10^-4^).

**Supplementary Table S6: GTEx database determined effect of rs1042173 polymorphism on serotonin transporter (*SLC6A4*) gene expression in different normal human tissues including sub-regions of brain**

| **Gene** | **Variant ID** | **Ref_Alt** | **Tissue** | **Total samples** | **Genotype** | | | **NES** | ***p*-value** | **m-value** |
| --- | --- | --- | --- | --- | --- | --- | --- | --- | --- | --- |
|  |  |  |  |  | **AA** | **AC** | **CC** |  |  |  |
| *SLC6A4* (ENSG00000108576.9) | rs1042173 (chr17_30197993_A_C_b38) | A_C | Brain - Amygdala | 129 | 48 | 54 | 27 | 0.021 | 0.8 | 0.29 |
|  |  |  | Brain - Anterior cingulate cortex (BA24) | 147 | 50 | 67 | 30 | 0.025 | 0.8 | 0.24 |
|  |  |  | Brain - Caudate (basal ganglia) | 194 | 74 | 88 | 32 | 0.094 | 0.3 | 0.10 |
|  |  |  | Brain - Cerebellar Hemisphere | 175 | 64 | 80 | 31 | 0.107 | 0.2 | 0.03 |
|  |  |  | Brain - Cerebellum | 209 | 77 | 88 | 44 | 0.106 | 0.2 | 0.18 |
|  |  |  | Brain - Cortex | 205 | 80 | 83 | 42 | 0.132 | 0.1 | 0.04 |
|  |  |  | Brain - Frontal Cortex (BA9) | 175 | 66 | 74 | 35 | 0.144 | 0.1 | 0.02 |
|  |  |  | Brain - Hippocampus | 165 | 70 | 67 | 28 | 0.059 | 0.5 | 0.09 |
|  |  |  | Brain - Hypothalamus | 170 | 67 | 71 | 32 | -0.111 | 0.2 | 0.57 |
|  |  |  | Brain - Nucleus accumbens (basal ganglia) | 202 | 75 | 90 | 37 | 0.027 | 0.8 | 0.23 |
|  |  |  | Brain - Putamen (basal ganglia) | 170 | 66 | 70 | 34 | -0.004 | 1 | 0.27 |
|  |  |  | Brain - Spinal cord (cervical c-1) | 126 | 45 | 57 | 24 | -0.054 | 0.7 | 0.49 |
|  |  |  | Brain - Substantia nigra | 114 | 41 | 46 | 27 | 0.032 | 0.8 | 0.20 |
|  |  |  | **Nerve - Tibial** | **532** | **163** | **221** | **81** | **-0.208** | **2.7 x 10^-6^** | **1.00** |
|  |  |  | **Esophagus - Muscularis** | **465** | **195** | **243** | **94** | **-0.252** | **7.6 x 10^-6^** | **1.00** |
|  |  |  | Whole Blood | 670 | 245 | 302 | 123 | 0.006 | 0.9 | 0.03 |

Alt, alternative allele; Ref, reference allele; NES, normalized effect size.

Significant eQTL-tissue pairs are presented in bold.

Total samples: represents the number of RNA-seq samples for which genotype data is available, NES: defined as slope of the linear regression representing eQTL effect sizes and is determined as the effect of the alternative allele (Alt) relative to the reference allele (Ref) in the human genome reference GRCh38/hg38, *p*-value: from a t-test that compares observed NES from single-tissue eQTL analysis to a null NES of 0, m-value: represents posterior probability that an eQTL effect exists in each tissue tested in the cross-tissue meta-analysis and ranges from 0 to 1 with thresholds of <0.1 and >0.9 for no effect and significant effect, respectively.

.

**Supplementary Table S7: GTEx database determined effect of rs6311 polymorphism on serotonin receptor (*HTR2A*) gene expression in different normal human tissues including sub-regions of brain**

| **Gene** | **Variant ID** | **Ref_Alt** | **Tissue** | **Total samples** | **Genotype** | | | **NES** | ***p*-value** | **m-value** |
| --- | --- | --- | --- | --- | --- | --- | --- | --- | --- | --- |
|  |  |  |  |  | **CC** | **CT** | **TT** |  |  |  |
| *HTR2A* (ENSG00000102468.10) | rs6311 (chr13_46897343_C_T_b38) | C_T | Brain - Amygdala | 129 | 45 | 66 | 18 | -0.061 | 0.3 | 0.00 |
|  |  |  | Brain - Anterior cingulate cortex (BA24) | 147 | 45 | 83 | 19 | 0.042 | 0.3 | 0.00 |
|  |  |  | Brain - Caudate (basal ganglia) | 194 | 61 | 104 | 29 | 0.149 | 0.05 | 0.00 |
|  |  |  | Brain - Cerebellar Hemisphere | 175 | 58 | 93 | 24 | -0.092 | 0.3 | 0.00 |
|  |  |  | Brain - Cerebellum | 209 | 66 | 114 | 29 | -0.059 | 0.5 | 0.00 |
|  |  |  | Brain - Cortex | 205 | 69 | 106 | 30 | -0.013 | 0.7 | 0.00 |
|  |  |  | Brain - Frontal Cortex (BA9) | 175 | 55 | 97 | 23 | -0.021 | 0.6 | 0.00 |
|  |  |  | Brain - Hippocampus | 165 | 50 | 90 | 25 | 0.021 | 0.7 | 0.00 |
|  |  |  | Brain - Hypothalamus | 170 | 49 | 96 | 25 | 0.063 | 0.3 | 0.00 |
|  |  |  | Brain - Nucleus accumbens (basal ganglia) | 202 | 64 | 106 | 32 | -0.026 | 0.7 | 0.00 |
|  |  |  | Brain - Putamen (basal ganglia) | 170 | 54 | 92 | 24 | 0.057 | 0.6 | 0.00 |
|  |  |  | Brain - Spinal cord (cervical c-1) | 126 | 34 | 74 | 18 | -0.093 | 0.3 | 0.00 |
|  |  |  | Brain - Substantia nigra | 114 | 35 | 62 | 17 | 0.109 | 0.1 | 0.00 |
|  |  |  | **Artery - Aorta** | **387** | **131** | **185** | **71** | **0.256** | **1.1 x 10^-6^** | **0.00** |
|  |  |  | **Testis** | **322** | **112** | **152** | **58** | **-0.469** | **9.2 x 10^-19^** | **1.00** |
|  |  |  | Whole Blood | 670 | NA | NA | NA | NA | NA | NA |

Alt, alternative allele; Ref, reference allele; NA, not available; NES, normalized effect size.

Significant eQTL-tissue pairs are presented in bold.

Total samples: represents the number of RNA-seq samples for which genotype data is available, NES: defined as slope of the linear regression representing eQTL effect sizes and is determined as the effect of the alternative allele (Alt) relative to the reference allele (Ref) in the human genome reference GRCh38/hg38, *p*-value: from a t-test that compares observed NES from single-tissue eQTL analysis to a null NES of 0, m-value: represents posterior probability that an eQTL effect exists in each tissue tested in the cross-tissue meta-analysis and ranges from 0 to 1 with thresholds of <0.1 and >0.9 for no effect and significant effect, respectively.

**Supplementary Table S8: GTEx database determined effect of rs17440378 polymorphism on serotonin receptor (*HTR2B*) gene expression in different normal human tissues including sub-regions of brain**

| **Gene** | **Variant ID** | **Ref_Alt** | **Tissue** | **Total samples** | **Genotype** | | | **NES** | ***p*-value** | **m-value** |
| --- | --- | --- | --- | --- | --- | --- | --- | --- | --- | --- |
|  |  |  |  |  | **CC** | **CT** | **TT** |  |  |  |
| *HTR2B* (ENSG00000135914.5) | rs17440378 (chr2_231114641_C_T_b38) | C_T | Brain - Amygdala | 129 | 94 | 35 | 0 | 0.120 | 0.5 | 0.20 |
|  |  |  | Brain - Anterior cingulate cortex (BA24) | 147 | 102 | 42 | 03 | -0.106 | 0.5 | 0.00 |
|  |  |  | Brain - Caudate (basal ganglia) | 194 | 137 | 55 | 02 | 0.153 | 0.2 | 0.06 |
|  |  |  | Brain - Cerebellar Hemisphere | 175 | 123 | 51 | 01 | 0.046 | 0.8 | 0.00 |
|  |  |  | Brain - Cerebellum | 209 | 146 | 60 | 03 | -0.401 | 2.9 x 10^-3^ | 0.00 |
|  |  |  | Brain - Cortex | 205 | 144 | 58 | 03 | 0.035 | 0.8 | 0.00 |
|  |  |  | Brain - Frontal Cortex (BA9) | 175 | 127 | 48 | 0 | -0.142 | 0.3 | 0.00 |
|  |  |  | Brain - Hippocampus | 165 | 118 | 45 | 02 | 0.033 | 0.8 | 0.00 |
|  |  |  | Brain - Hypothalamus | 170 | 122 | 47 | 01 | 0.123 | 0.4 | 0.06 |
|  |  |  | Brain - Nucleus accumbens (basal ganglia) | 202 | 142 | 58 | 02 | 0.113 | 0.4 | 0.00 |
|  |  |  | Brain - Putamen (basal ganglia) | 170 | 121 | 48 | 01 | -0.028 | 0.8 | 0.003 |
|  |  |  | Brain - Spinal cord (cervical c-1) | 126 | 93 | 33 | 0 | 0.396 | 0.03 | 0.80 |
|  |  |  | Brain - Substantia nigra | 114 | 84 | 28 | 02 | 0.262 | 0.2 | 0.25 |
|  |  |  | **Esophagus - Gastroesophageal Junction** | **330** | **235** | **90** | **05** | **0.600** | **6.7 x 10^-14^** | **1.00** |
|  |  |  | **Esophagus - Muscularis** | **465** | **339** | **118** | **08** | **0.593** | **7.9 x 10^-20^** | **1.00** |
|  |  |  | **Artery - Aorta** | **387** | **276** | **105** | **06** | **0.575** | **1.6 x 10^-12^** | **1.00** |
|  |  |  | **Lung** | **515** | **366** | **138** | **11** | **0.319** | **2.5 x 10^-7^** | **1.00** |
|  |  |  | **Heart - Atrial Appendage** | **372** | **260** | **103** | **09** | **-0.302** | **. x 10^-1^** | **0.00** |
|  |  |  | **Artery - Coronary** | **213** | **155** | **55** | **03** | **0.494** | **9.4 x 10^-6^** | **1.00** |
|  |  |  | **Testis** | **322** | **225** | **90** | **07** | **0.333** | **1.5 x 10^-5^** | **0.97** |
|  |  |  | Whole Blood | 670 | NA | NA | NA | NA | NA | NA |

Alt, alternative allele; Ref, reference allele; NA, not available; NES, normalized effect size.

Significant eQTL-tissue pairs are presented in bold.

Total samples: represents the number of RNA-seq samples for which genotype data is available, NES: defined as slope of the linear regression representing eQTL effect sizes and is determined as the effect of the alternative allele (Alt) relative to the reference allele (Ref) in the human genome reference GRCh38/hg38, *p*-value: from a t-test that compares observed NES from single-tissue eQTL analysis to a null NES of 0, m-value: represents posterior probability that an eQTL effect exists in each tissue tested in the cross-tissue meta-analysis and ranges from 0 to 1 with thresholds of <0.1 and >0.9 for no effect and significant effect, respectively.

**Supplementary Table S9: Primer sequences, PCR amplification and assay details for genotyping of studied polymorphisms in *5HTT,* *HTR2A* and *HTR2B* genes**

| **Polymorphisms** | **Primers** | **Annealing temperature (Product size)** | **Genotyping approach** | **Interpretation of genotypes^1^** | **Reference** |
| --- | --- | --- | --- | --- | --- |
| ***5HTT*** | | | | | |
| HTTLPR | Forward: 5՛ ACTCCCTGTACCCCTCCTAG3՛  Reverse: 5՛ GGATACTGCGAGGGGTGCA3՛ | 62^o^C (179bp/222bp) | PCR | - S/S = 179bp - S/L = 179bp, 222bp - L/L = 222bp | [^1^](#_ENREF_1) |
| rs255331 in HTTLPR polymorphism | Forward: 5՛ ACTCCCTGTACCCCTCCTAG3՛  Reverse: 5՛ GGATACTGCGAGGGGTGCA3՛ | 62^o^C (179bp/222bp) | PCR-RFLP assay using Msp1 | - S/S = 179bp - S/L_G_ = 82bp, 140bp, 179bp - L_G_/L_G_ = 82bp, 140bp - S/L_A_ = 179bp, 222bp - L_A_/ L_G_ = 82bp, 140bp, 222bp - L_A_/ L_A_ = 222bp | [^2^](#_ENREF_2) |
| STin2 | Forward: 5՛ GTCAGTATCACAGGCTGCGAG3՛  Reverse: 5՛ TGTTCCTAGTCTTACGCCAGTG3՛ | 62^o^C (265bp/291bp) | PCR | - 12R/12R = 291bp - 12R/10R = 265bp, 291bp - 10R/10R = 265bp | This study |
| 3՛UTR (rs1042173) | Forward: 5՛GCGTAGGAGAGAACAGGGAT3՛  Reverse: 5՛ CTGTAAAGGAAAGTGTGTGGCT3՛ | 62^o^C (391bp) | PCR-RFLP assay using Mse1 | - G/G = 391bp - G/T = 125bp, 266bp, 391bp - T/T = 125bp, 266bp | [^3^](#_ENREF_3) |
| ***HTR2A*** | | | | | |
| rs6311 | Forward: 5՛ TGGCCTTTTGTGCAGATTCCCA3՛  Reverse: 5՛ CTAGCCACCCTGAGCCTATG3՛ | 62^o^C (207bp) | PCR-RFLP assay using Msp1 | - C/C = 81bp, 126bp - C/T = 81bp, 126bp, 207bp - T/T = 207bp | This study |
| ***HTR2B*** | | | | | |
| rs17440378 | Custom designed iPLEX SNP genotyping assay | - | iPLEX assay and the MassARRAY system | - | - |

^1^The genotypes for tri-allelic locus of rs25531 in HTTLPR were described based on the functional activity as low (S/S, L_G_/S, L_G_/L_G_), intermediate (L_A_/S, L_A_/L_G_) and High (L_A_/L_A_).

bp, base pairs; ^o^C, centigrade; PCR, polymerase chain reaction; RFLP, restriction fragment length polymorphism.

**References**

1 Lesch, K. P. *et al.* Association of anxiety-related traits with a polymorphism in the serotonin transporter gene regulatory region. *Science (New York, N.Y.)* **274**, 1527-1531 (1996).

2 Wendland, J., Martin, B., Kruse, M., Lesch, K. & Murphy, D. Simultaneous genotyping of four functional loci of human SLC6A4, with a reappraisal of 5-HTTLPR and rs25531. *Mol. Psychiatry* **11**, 224 (2006).

3 Battersby, S. *et al.* Presence of multiple functional polyadenylation signals and a single nucleotide polymorphism in the 3′ untranslated region of the human serotonin transporter gene. *J. Neurochem.* **72**, 1384-1388 (1999).

.

**
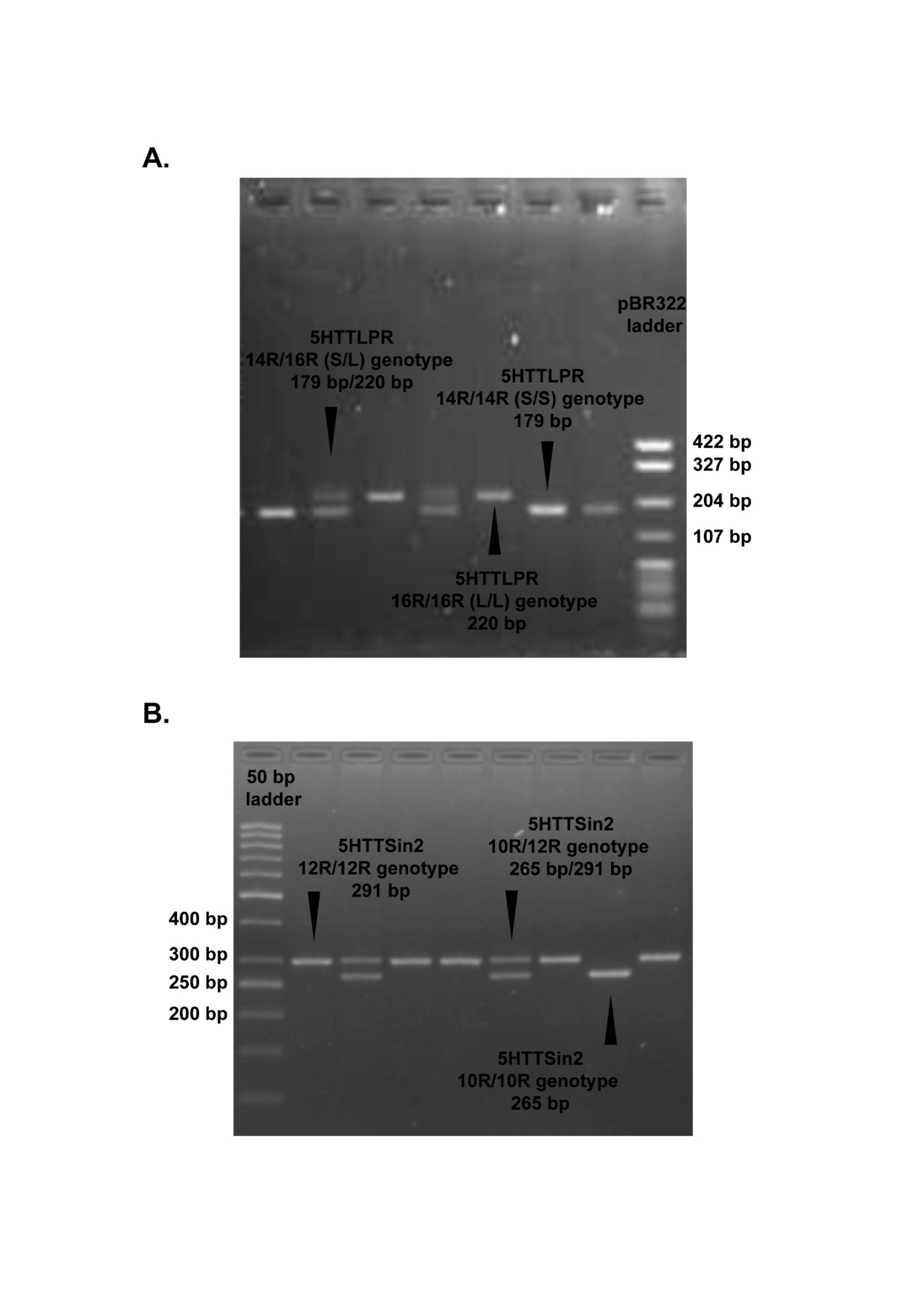
**

**Supplementary Figure S3: PCR based genotyping of 5-HTTLPR and STin2 polymorphisms of *SLC6A4* gene. A)** 44bp VNTR bi-allelic 5-HTTLPR polymorphism. **B)** 17bp VNTR STin2 polymorphism.

**
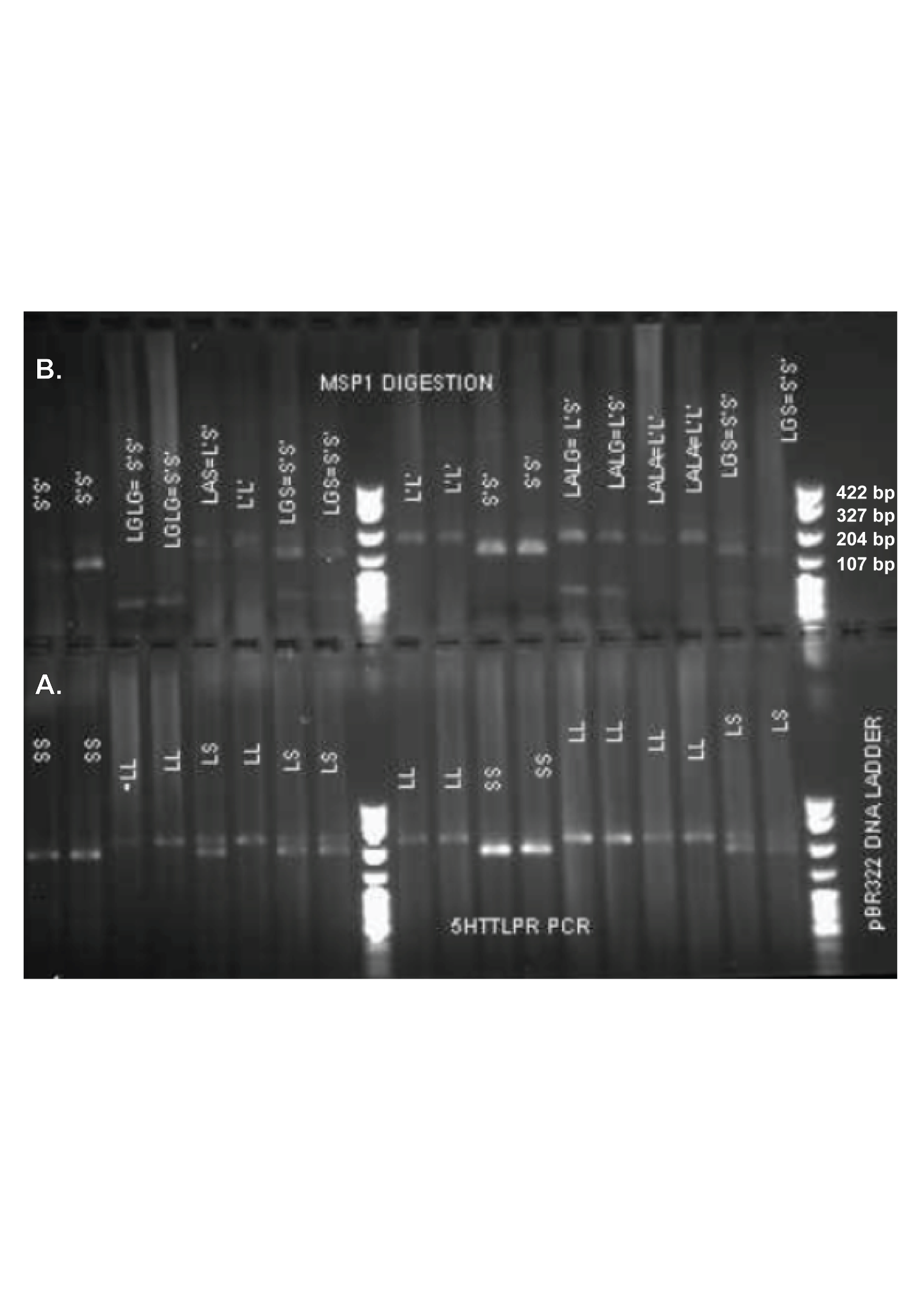
**

**Supplementary Figure S4: PCR-RFLP based genotyping of tri-allelic rs25531 in 5-HTTLPR polymorphism of *SLC6A4* gene. A)** PCR amplification of 5-HTTLPR region containing rs25531SNP **B)** RFLP analysis and genotypes of rs25531 in 5-HTTLPR after MspI restriction digestion.

**
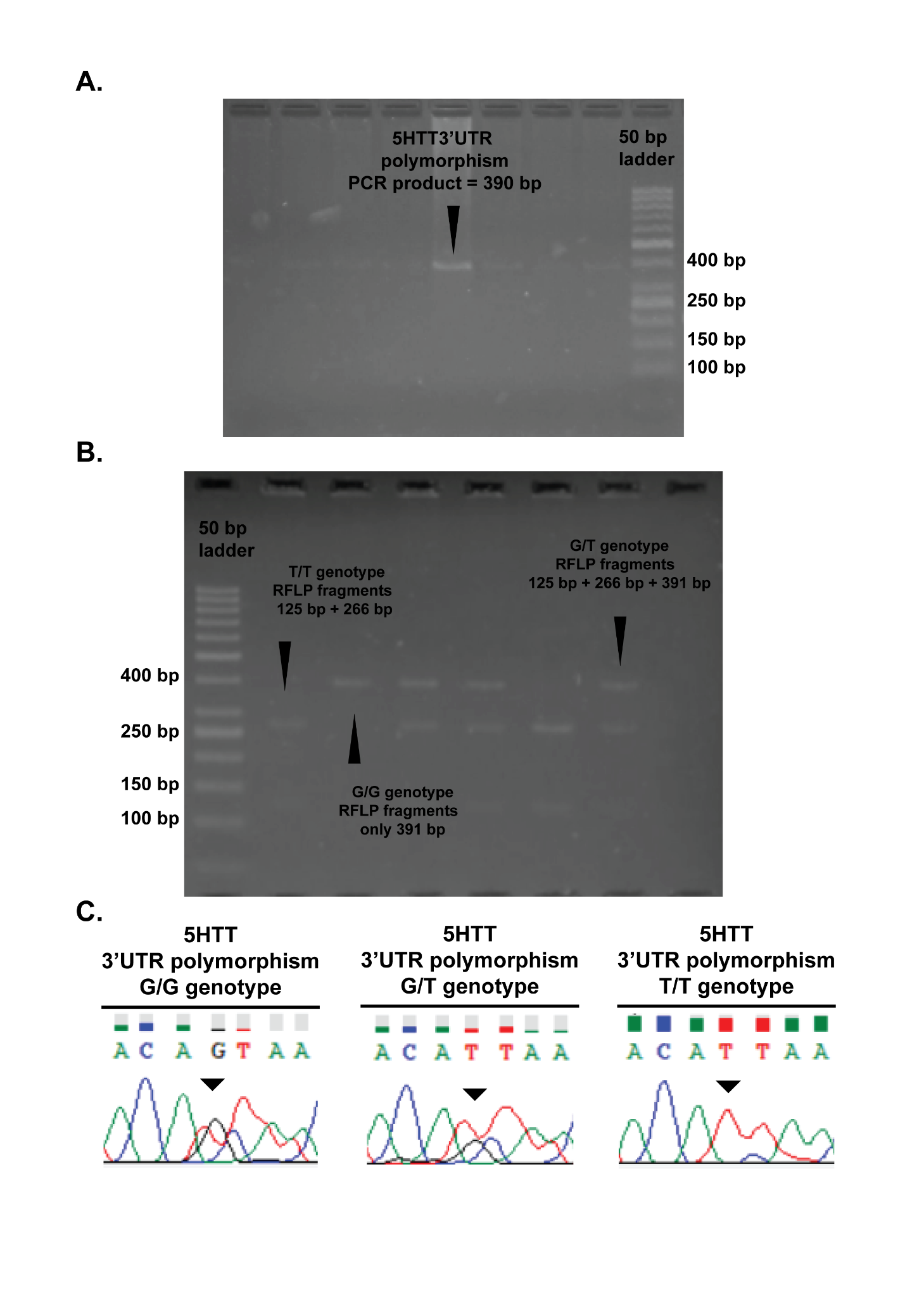
**

**Supplementary Figure S5: PCR-RFLP based genotyping and Sanger confirmation of *SLC6A4* (*5-HTT)* 3′UTR polymorphism (rs1042173). A)** PCR amplification. **B)** RFLP analysis. **C)** Sanger sequencing validation of PCR-RFLP genotyping.

**
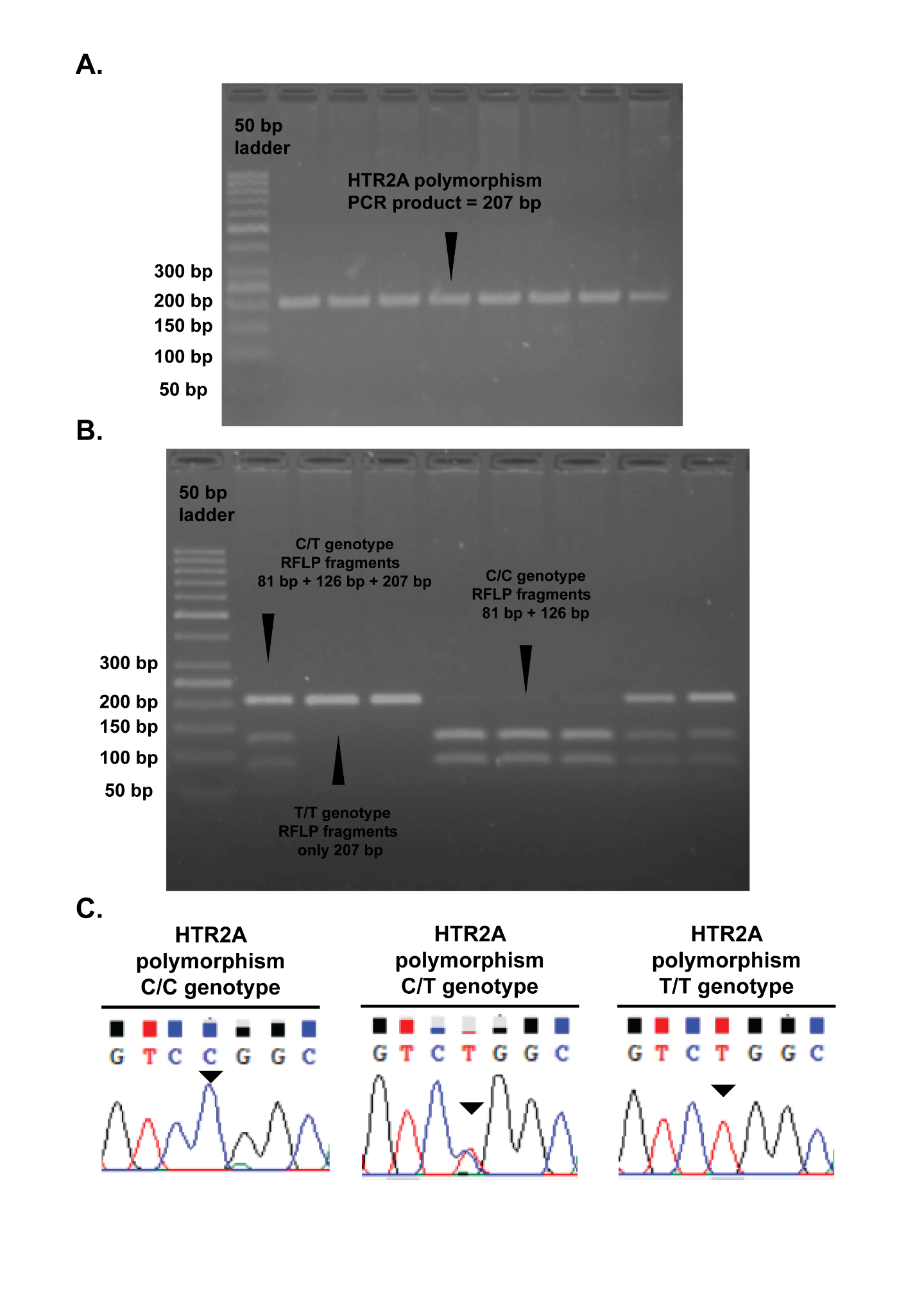
**

**Supplementary Figure S6: PCR-RFLP based genotyping and Sanger confirmation of *HTR2A* polymorphism (rs6311). A)** PCR amplification. **B)** RFLP analysis. **C)** Sanger sequencing validation of PCR-RFLP genotyping.
